# ROCK inhibitor combined with Ca^2+^ controls the myosin II activation and optimizes human nasal epithelial cell sheets

**DOI:** 10.1038/s41598-020-73817-3

**Published:** 2020-10-08

**Authors:** Yoshiyuki Kasai, Tsunetaro Morino, Eri Mori, Kazuhisa Yamamoto, Hiromi Kojima

**Affiliations:** Department of Otorhinolaryngology, Jikei University School of Medicine, 3-25-8 Nishi-Shinbashi, Minato-ku, Tokyo, 105-8461 Japan

**Keywords:** Regeneration, Post-translational modifications, Translational research

## Abstract

The proliferation and differentiation of cultured epithelial cells may be modified by Rho-associated kinase (ROCK) inhibition and extracellular Ca^2+^ concentration. However, it was not known whether a combination would influence the behavior of cultured epithelial cells through changes in the phosphorylation of non-muscle myosin light chain II (MLC). Here we show that the combination of ROCK inhibition with Ca^2+^ elevation regulated the phosphorylation of MLC and improved both cell expansion and cell–cell adhesion during the culture of human nasal mucosal epithelial cell sheets. During explant culture, Ca^2+^ enhanced the adhesion of nasal mucosal tissue, while ROCK inhibition downregulated MLC phosphorylation and promoted cell proliferation. During cell sheet culture, an elevation of extracellular Ca^2+^ promoted MLC phosphorylation and formation of cell–cell junctions, allowing the harvesting of cell sheets without collapse. Moreover, an in vitro grafting assay revealed that ROCK inhibition increased the expansion of cell sheets three-fold (an effect maintained when Ca^2+^ was also elevated), implying better wound healing potential. We suggest that combining ROCK inhibition with elevation of Ca^2+^ could facilitate the fabrication of many types of cell graft.

## Introduction

Previous studies have attempted to optimize the fabrication of epithelial cell grafts by supplementing the culture medium with agents such as cholera toxin^1^, epidermal growth factor^2^, amphotericin B^3^, insulin and hydrocortisone^4^. As a result of advances in cell culture techniques, many types of epithelial cell can be expanded in vitro to form cell sheets, which have been used for regeneration of the cornea^5^, esophagus^6–8^ and middle ear^9^. The selection of agents for optimization of cell culture is based on cell behaviors such as proliferation and differentiation. A key modulator of cell behavior is the non-muscle myosin light chain II (MLC), which is regulated by phosphorylation and has been implicated in cell migration, proliferation and differentiation^10–12^. Thus, controlling the level of phosphorylated MLC is considered important for the successful fabrication of cell grafts.

Rho signaling induced by Rho-associated kinase (ROCK) results in the phosphorylation and activation of MLC and hence muscle contraction^13–15^. Y-27632 is a ROCK inhibitor (ROCKi) that was originally developed as a muscle relaxant^16^ but subsequently shown to enhance the proliferation of many cell types including human embryonic stem cells and keratinocytes^17,18^. Additionally, Y-27632 has been co-injected with cultured corneal endothelial cells into the anterior chamber of the eye to regenerate the corneal endothelium in patients with bullous keratopathy^19^. Currently, a ROCKi is considered almost a necessity for cell expansion because of its anti-apoptotic and proliferative effects^17^.

Ionized calcium (Ca^2+^) induces calmodulin-mediated signaling that regulates the activity of MLC^20,21^. Ca^2+^ is also recognized as a differentiation-inducing factor for epithelial cells and is essential for cell–cell adhesion via cadherin^22–25^. Culture medium containing low levels of Ca^2+^ prevents the differentiation of epithelial cells and promotes cell expansion^26,27^. Remarkably, Zhang et al. reported that the combination of a ROCKi with low-Ca^2+^ medium resulted in a 10^12^-fold expansion in epithelial cell number^28^. Therefore, addition of a ROCKi and alteration of the extracellular Ca^2+^ concentration are two methods that can be used to regulate the MLC activity, proliferation and differentiation of epithelial cells.

We recently succeeded in treating patients with severe middle ear disease by surgically transplanting nasal mucosal cell sheets into the middle ear^9^. The cell sheets were fabricated using a two-step process involving explant culture (proliferation) and cell sheet culture (differentiation) in keratinocyte culture medium (KCM)^29,30^. The transplant surgery required up to 9 nasal mucosal cell sheets per patient, which would necessitate a highly efficient culture protocol to generate enough autologous cell sheets from a single specimen of resected nasal mucosa. Analyzing the effects of various agents on cell behavior during the two stages of culture would provide important information regarding how to optimize cell proliferation and differentiation during the fabrication of cell sheets.

In this study, we examined the effects of co-administering different concentrations of Y-27632 and Ca^2+^ on cell proliferation and differentiation during explant culture and cell sheet culture. Furthermore, we analyzed the expressions of various proteins, particularly MLC and its phosphorylated forms, to better understand the effects of these agents on cell phenotype and behavior. We also investigated the wound healing potential of nasal mucosal cell sheets fabricated using optimized levels of Y-27632 and Ca^2+^.

## Results

### ROCK inhibition and Ca^2+^ are important for explant culture

**Figure 1:**
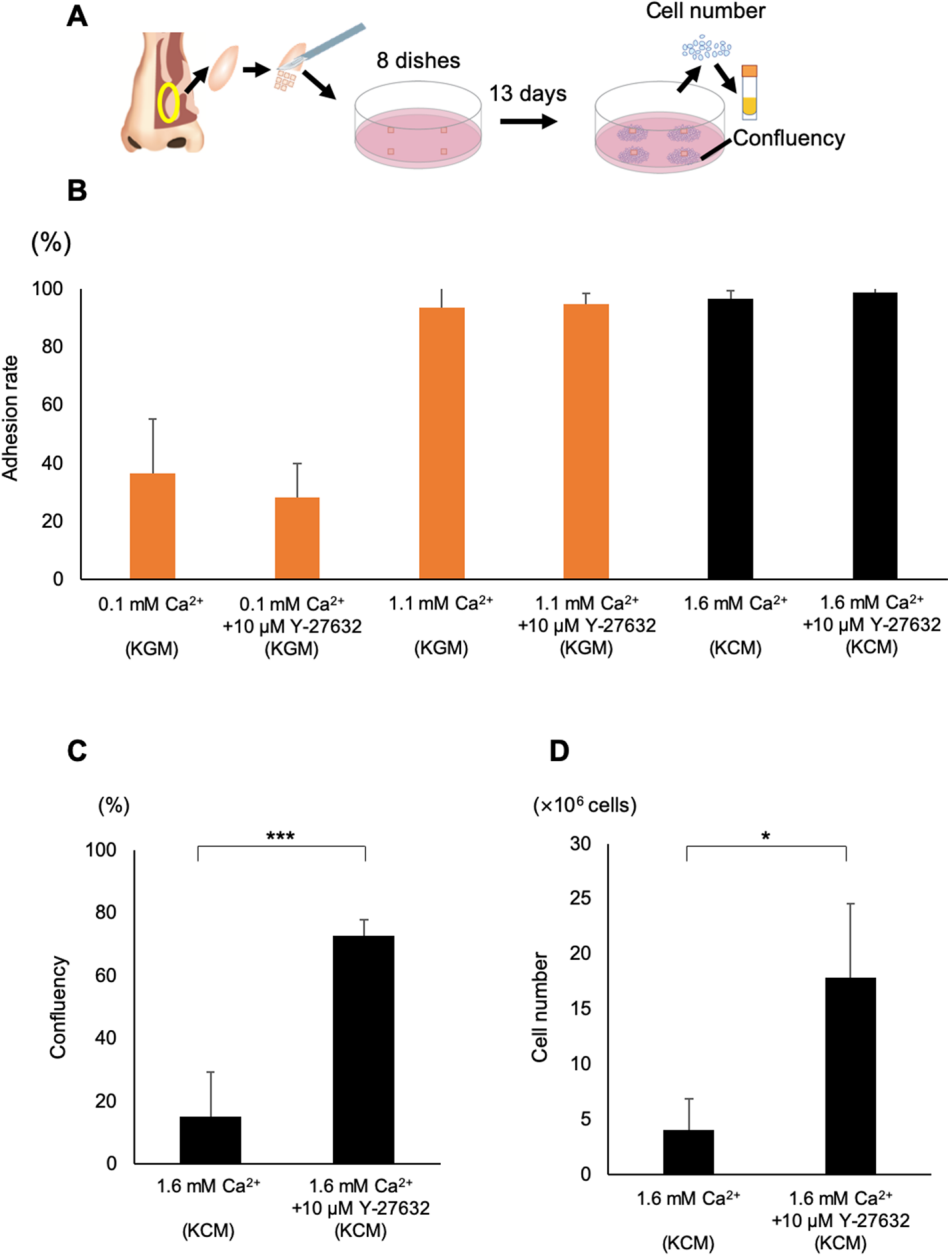
Inhibition of ROCK promotes explant culture in KCM. (**A**) Schematic representation of the study protocol for explant culture. Human nasal tissues were resected, placed in 8 culture dishes and cultured for 13 days. Cells were collected using trypsin–EDTA and then preserved at − 190 °C. (**B**) Adhesion rates for explant cultures in KGM containing 0.1 mM Ca^2+^ (0.1 mM Ca^2+^), KGM containing 10 μM Y-27632 (0.1 mM Ca^2+^  + 10 μM Y-27632), KGM containing 1.1 mM Ca^2+^ (1.1 mM Ca^2+^), KGM containing 10 μM Y-27632 and 1.1 mM Ca^2+^ (1.1 mM Ca^2+^  + 10 μM Y-27632), KCM containing 1.6 mM Ca^2+^ (1.6 mM Ca^2+^) and KCM containing 1.6 mM Ca^2+^ and 10 μM Y-27632 (1.6 mM Ca^2+^  + 10 μM Y-27632). Values are expressed as the mean ± SEM (*n* = 3). (**C**) Cell confluence in a 60-mm dish for cells cultured in KCM containing 1.6 mM Ca^2+^ (*n* = 5) or KCM containing 1.6 mM Ca^2+^  + 10 μM Y-27632 (*n* = 6). (**D**) Cell number counted from 8 dishes of cells cultured using KCM containing 1.6 mM Ca^2+^ or KCM containing 1.6 mM Ca^2+^  + 10 μM Y-27632. Values are expressed as the mean ± SEM. *P < 0.05, ***P < 0.001.

So that our cell sheets would have the potential for future use in a clinical setting, we consulted with the Pharmaceuticals and Medical Devices Agency of Japan (which has a similar role to the US Food and Drug Administration) to ensure that we used high-grade reagents and materials. First, we confirmed successful explant culture in KCM supplemented with 5% autologous human serum (KCM-HS) using our previously reported method^30^ (Fig. 1A). Changing some of the reagents and materials (from a rat-derived collagen dish to a Primaria dish or from Dulbecco’s modified Eagle medium (DMEM)/F-12 containing 4-(2-hydroxyethyl)-1-piperazineethanesulfonic acid [HEPES] to DMEM/F-12 without HEPES) did not result in any significant effects on cell number or cell expansion area (*n* = 5, data not shown). Since fetal bovine serum (FBS) is readily available and avoids the processes needed to obtain autologous human serum (including venipuncture, centrifugation and filtration), one of our objectives was to optimize the culture conditions to allow us to use FBS. However, nasal cells reached a confluency of only 15 ± 5% (*n* = 4) when cultured in KCM containing 10% fetal bovine serum (FBS), which was significantly lower than that achieved with KCM-HS (38 ± 14%, *n* = 5; *P* < 0.05, Fig. S1A). As a result, the number of cells obtained using KCM-FBS (4.0 ± 2.9 × 10^6^, *n* = 4) was also significantly lower than that obtained using KCM-HS (13.6 ± 7.1 × 10^6^, *n* = 7; *P* < 0.01; Fig. S1B). Although we examined three different types of γ-irradiated FBS, satisfactory cell proliferation was not achieved with any of these (data not shown).

Since low-Ca^2+^ medium (~ 1 mM) is useful for expanding epithelial cells^22^, we investigated whether lowering the Ca^2+^ concentration might improve cell confluency when explant culture was performed using FBS. However, the tissue adhesion rate was only 36% (*n* = 3) when explant culture was performed in Keratinocyte Growth Medium (KGM) containing 0.1 mM Ca^2+^ (analyzed in experiments using Arsenazo III; *n* = 2). Notably, the tissue adhesion rate reached approximately 94% (*n* = 3) when the Ca^2+^ concentration was increased to 1.1 mM (KGM supplemented with additional Ca^2+^) and 99% (*n* = 3) when 1.6 mM Ca^2+^ (KCM-FBS) was used (Fig. 1B). These results illustrate that tissue adhesion could be achieved successfully during explant culture when a Ca^2+^ concentration of 1.1–1.6 mM was used, whereas very low levels of Ca^2+^ were not suitable for explant culture.

Since inhibition of ROCK is known to stimulate cell proliferation, next we investigated whether the addition of 10 μM Y-27632 would improve confluency and cell numbers when explant culture was performed in 1.6 mM Ca^2+^ (KCM-FBS). Cell confluence after 13 days of cultivation in medium containing 1.6 mM Ca^2+^ and 10 μM Y-27632 was 73 ± 11% (*n* = 4), which was significantly higher than that achieved in the absence of Y-27632 (15 ± 5%, *n* = 4; *P* < 0.001, Fig. 1C). Furthermore, culture in medium containing 1.6 mM Ca^2+^ and 10 μM Y-27632 enabled the collection of 1.8 ± 6.8 × 10^7^ cells (*n* = 6), which was significantly more than that in the absence of Y-27632 (*P* < 0.0001, Fig. 1D). Notably, the addition of 10 μM Y-27632 to the culture medium (1.6 mM Ca^2+^) allowed a minimum of 8.9 × 10^6^ cells to be collected; since 8.8 × 10^5^ nasal cells are needed for each cell sheet, the use of Y-27632 allowed enough cells to be obtained for at least 10 cell sheets.

### Effect of ROCK inhibition on nasal epithelial cell outgrowth patterns

**Figure 2:**
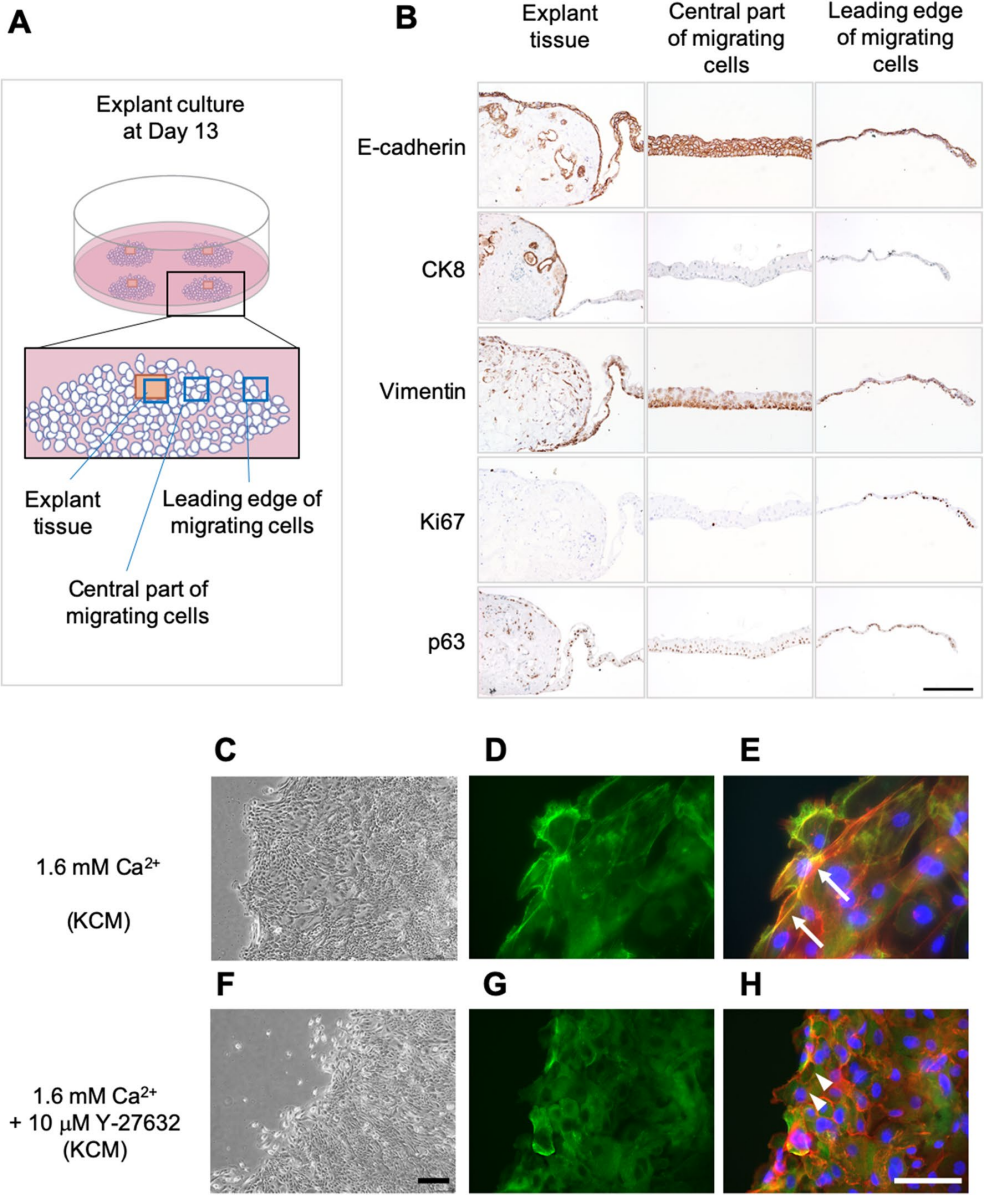
Protein expression during explant culture. (**A**) Schematic diagram illustrating the positions of the tissues shown in B (explant tissue, central part of the migrating cells and leading edge of the migrating cells). (**B**) Immunohistological evaluation of the expressions of E-cadherin, CK8, vimentin and Ki67 in the explant tissue (left column), central part of the migrating cells (center column), and leading edge of the migrating cells (right column). (**C**) Phase-contrast microscopy images of the peripheral region of outgrowing cells cultured in normal KCM containing 1.6 mM Ca^2+^. (**D**) Immunohistological detection of P-MLC (green) at day 13 of explant culture in normal KCM containing 1.6 mM Ca^2+^. (**E**) Image in C merged with images stained using phalloidin (red) and DAPI (blue). White arrows indicate stress fibers with phosphorylated MLC. (F) Phase-contrast microscopy images of the peripheral region of outgrowing cells cultured in KCM containing 1.6 mM Ca^2+^  + 10 μM Y-27632. (**G**) Immunolocalization of P-MLC (green) at day 13 of explant culture in KCM containing 1.6 mM Ca^2+^  + 10 μM Y-27632. (**H**) Image in F merged with images stained using phalloidin (red) and DAPI (blue). White arrowheads indicate actin depolymerization. Scale bars = 200 μm (**B**), 300 μm (**C**,**F**) and 100 μm (**D**,**E**,**G**,**H**).

To better evaluate tissue adhesion during explant culture, we performed our explant culture in temperature-responsive cell culture dishes using KCM-FBS (1.6 mM Ca^2+^). After cell outgrowth was observed, we carefully detached the tissue as a cell sheet to make paraffin-embedded sections that were subjected to immunohistology (Fig. 2A,B). In addition, non-cultured nasal mucosal tissues were used as controls (Fig. S2). In native tissue (before explant culture), E-cadherin (an epithelial marker) was expressed in epithelial cells only, cytokeratin-8 (CK8, a marker of differentiated nasal epithelium) was expressed in the upper layer of epithelial cells, vimentin (a marker of fibroblast/epithelial-mesenchymal transition) was expressed only in fibroblasts, Ki67 (a proliferation marker) was expressed sparsely in the epithelial layer, and p63 (a marker of stem/progenitor cells) was expressed on the basal side of epithelial cells. Immunodetection of E-cadherin in the explant culture at day 13 showed that connective tissue was present under the epithelial cells and had adhered before epithelial cell adhesion/migration. CK8, an intermediate filament found in nasal epithelial cells, was expressed in the tissue area but not in migrating cells. Vimentin was expressed not only in fibroblasts in the connective tissue but also in migrating epithelial cells. Interestingly, the proliferation marker Ki67 was observed in leading-edge cells rather than within the tissue area. These results indicate that cells in the leading edge are important for cell expansion.

In order to observe the leading edge in more detail, we analyzed its behavior using phase-contrast microscopy and time-lapse differential interference microscopy. In normal KCM (1.6 mM Ca^2+^), the cells migrated collectively (Fig. 2C). Although most cells moved outward, an interesting observation was that part of the cell cluster at the edge moved inward (Movie 1). Since this inward movement was considered actomyosin behavior, we analyzed the distribution of phospho-myosin light chain II (P-MLC) and actin stress fibers (Fig. 2D,E). As expected, P-MLC existed at the edges of each cell in normal KCM, and stress fibers were observed at cell–cell sites along with P-MLC (Fig. 2E). By contrast, when 10 µM Y-27632 was added, epithelial cells only moved toward the periphery as an outgrowth, and some of the cells migrated individually (Fig. 2F, Movie 2). In the presence of 10 µM Y-27632, P-MLC existed in the cytoplasm (Fig. 2G), and actin depolymerization was observed (Fig. 2H).

### Optimization of cell sheet culture conditions

**Figure 3:**
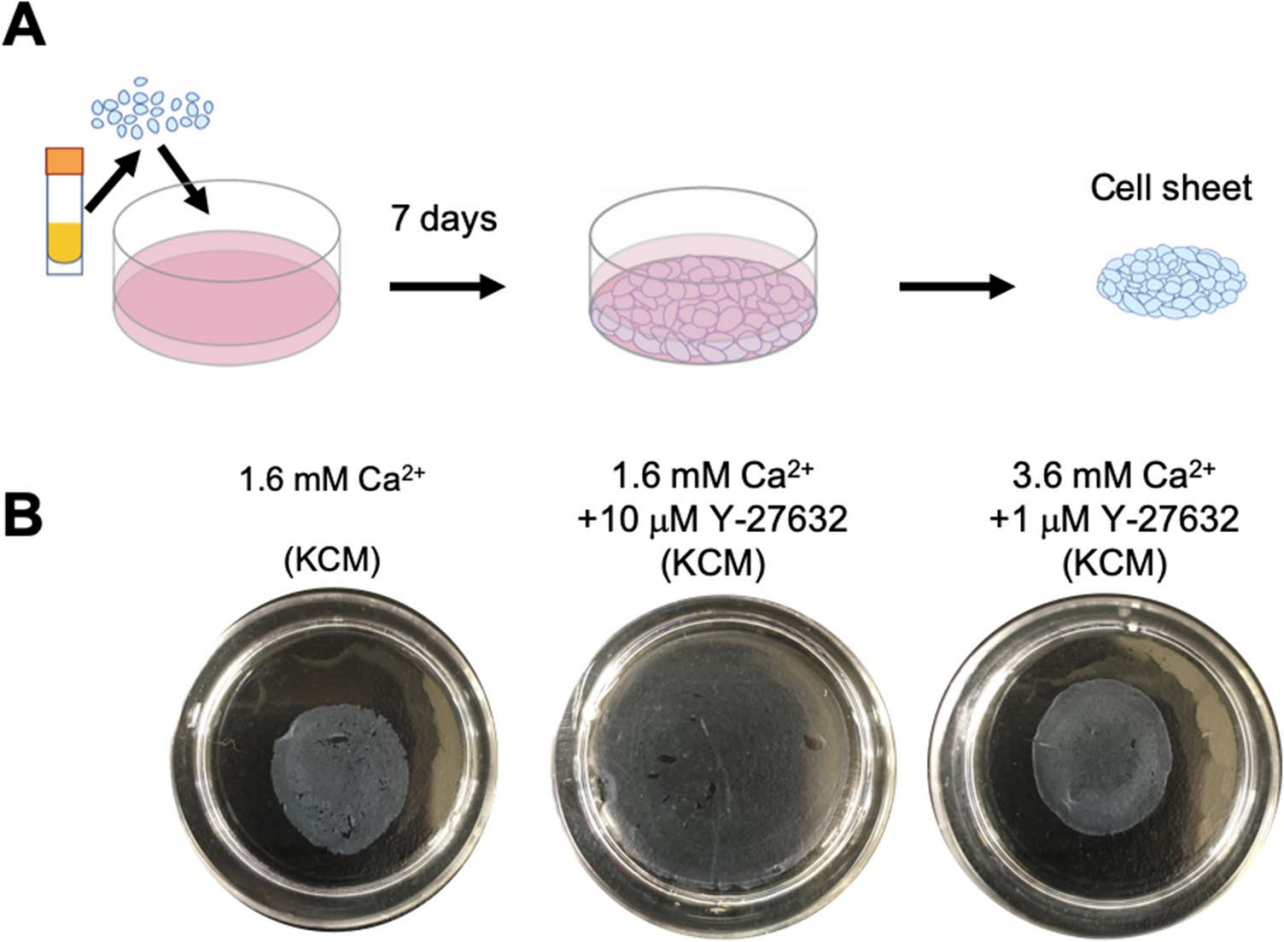
Macroscopic features of cell sheets cultured under three experimental conditions. (**A**) Thawed cells were seeded on a temperature-responsive cell culture dish at a density of 1.0 × 10^5^ cells/cm^2^ and then cultivated for 7 days. (**B**) Representative images showing cell sheets cultured in KCM containing 1.6 mM Ca^2+^, KCM containing 1.6 mM Ca^2+^  + 10 μM Y-27632, or KCM containing 3.6 mM Ca^2+^  + 1 μM Y-27632.

Freeze-thawed cells were able to form a nasal mucosal cell sheet when cultured in KCM-HS (Figs. 3A, S3A), as reported in our previous article^30^. However, defects were observed in the cell sheets when they were cultured in normal KCM-FBS containing 1.6 mM Ca^2+^ (Fig. 3B). Based on the results of the explant culture experiments, we initially evaluated whether the addition of 10 µM Y-27632 would improve cell sheet culture in KCM-FBS. However, culture in KCM-FBS containing 1.6 mM Ca^2+^ and 10 µM Y-27632 generated cell sheets that were structurally loose (i.e., did not show shrinkage behavior after detachment from the dish) and contained large defects (Fig. 3B). Therefore, we examined whether removal of Y-27632 during cell sheet culture or alteration of its concentration would improve cell sheet morphology. Although excluding Y-27632 from day 0, 3 or 5 produced cell sheets without defects in some cases, defects remained in other cases (Fig. S3B). Reducing the concentration of Y-27632 also led to some improvement, with 1 μM Y-27632 yielding better results than 100 nM or 2.5 μM Y-27632; nevertheless, some defects were still observed (Fig. S3C).

Since the best results were obtained using 1 μM Y-27632, we next examined whether increasing the Ca^2+^ concentration from 1.6 mM to either 2.6 mM, 3.6 mM or 5.6 mM would improve cell–cell adhesion and yield cell sheets that did not develop defects or shrink after detachment from the dish following culture in medium containing 1 μM Y-27632. Medium containing 1 μM Y-27632 and either 2.6 mM Ca^2+^ or 3.6 mM Ca^2+^ produced cell sheets that were easily detached and shrank spontaneously without the development of defects (Fig. 3B, S3D), and the effects of increasing the Ca^2+^ concentration to 3.6 mM were reproducible irrespective of the condition of the original tissue (Fig. S4). Detachable cell sheets were also fabricated in medium containing 1 μM Y-27632 and 5.6 mM Ca^2+^ (Fig. S3D), but numerous crystals and floating dead cells were observed (data not shown). Structural loosening of the cell sheet was observed when it was cultured in medium containing 10 μM Y-27632 and 3.6 mM Ca^2+^, and exclusion of Y-27632 from KCM containing 3.6 mM Ca^2+^ resulted in a cell sheet with many defects (Fig. S3E,F). Based on the above findings, it was concluded that optimal culture of nasal cell sheets was achieved using KCM-FBS containing 1 μM Y-27632 and 3.6 mM Ca^2+^.

### Phosphorylation of MLC during cell sheet culture

**Figure 4:**
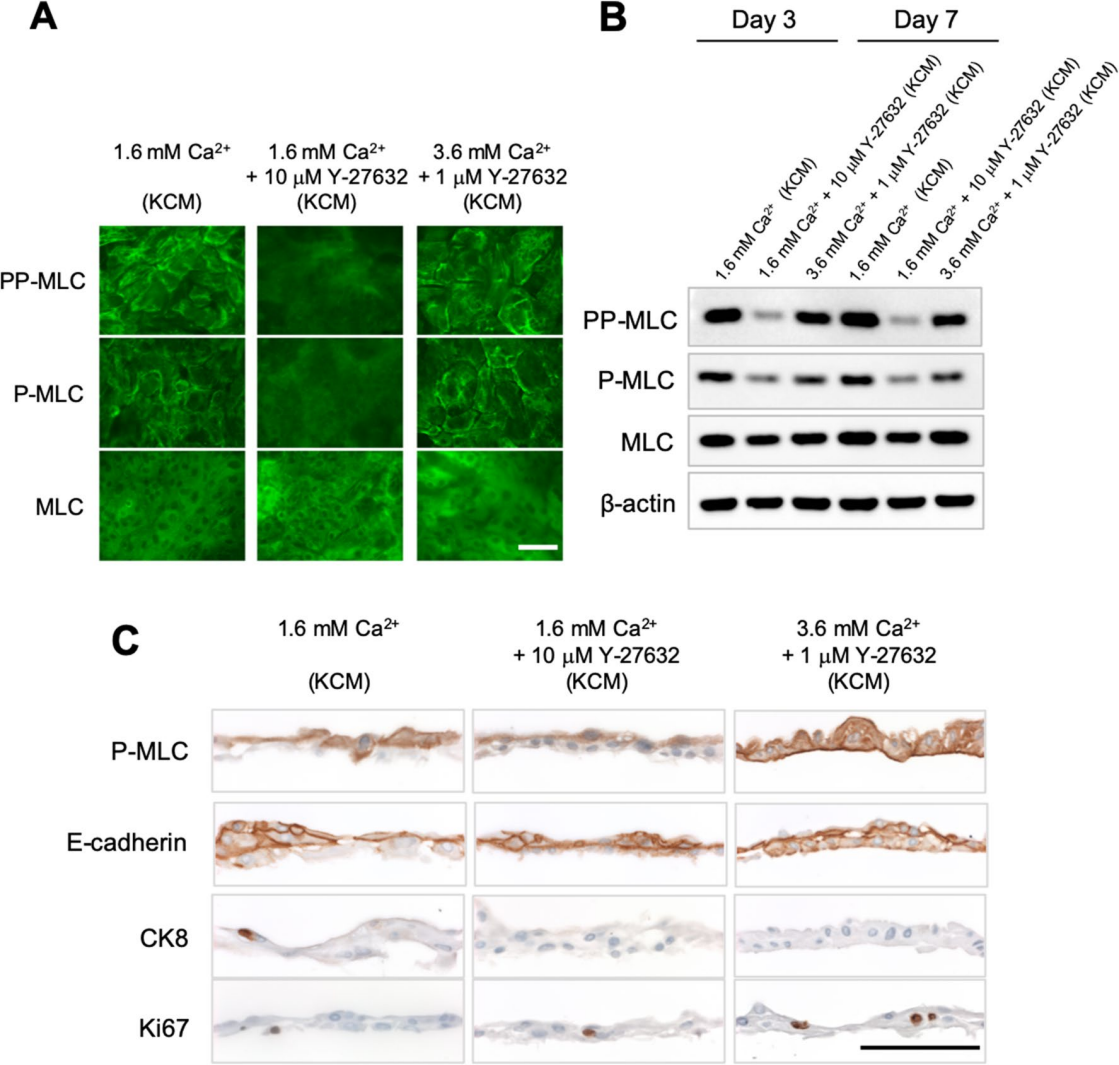
Protein expression in cell sheets cultured in KCM containing 1.6 mM Ca^2+^, KCM containing 1.6 mM Ca^2+^  + 10 μM Y-27632, or KCM containing 3.6 mM Ca^2+^  + 1 μM Y-27632. (**A**) Immunofluorescence images showing the expression of MLC, P-MLC and PP-MLC at day 7 of cell sheet culture. (**B**) Western blot analysis of MLC, P-MLC, PP-MLC and β-actin expression at day 3 and day 7 of cell sheet culture. (**C**) Immunohistological evaluation of harvested cell sheets. Scale bar = 100 μm (**A**,**C**).

To examine whether the effects of Y-27632 and Ca^2+^ on the shrinkage behavior of the cell sheet was related to MLC phosphorylation, we performed immunofluorescence and Western blotting to detect MLC, P-MLC (Ser19) and double-phospho-myosin light chain II (Thr18, Ser19; PP-MLC). Immunofluorescence experiments showed that PP-MLC and P-MLC were present at the periphery of each cell in medium containing 1.6 mM Ca^2+^ (normal KCM-FBS) but were barely detectable at the cell–cell interfaces in medium containing 1.6 mM Ca^2+^ and 10 µM Y-27632 (Fig. 4A). Interestingly, increasing the Ca^2+^ concentration from 1.6 mM to 3.6 mM recovered the phosphorylation of MLC at the cell periphery in the presence of 1 μM Y-27632 (Fig. 4A) but not in the presence of 10 μM Y-27632 (Fig. S5). The results of Western blotting experiments showed that phosphorylation of MLC was downregulated by the addition of Y-27632 to medium containing 1.6 mM Ca^2+^ but was recovered at both day 3 and day 7 when the Ca^2+^ concentration was increased from 1.6 mM to 3.6 mM (Fig. 4B; full-length Western blot in Fig. S7). Thus, the Western blot findings support the immunohistology data. In additional experiments, P-MLC was found to be weakly expressed in 0.1 mM Ca^2+^ (KGM), and the expression of P-MLC was downregulated when Y-27632 was added, indicating that ROCK signaling might be active under these conditions (Fig. S5). Furthermore, increasing the level of Ca^2+^ to ~ 2.2 mM (by adding extra Ca^2+^ to KGM) led to P-MLC being expressed at the periphery of each cell, suggesting that it might be promoting cell–cell adhesion (Fig. S5). These results show that an increase in extracellular Ca^2+^ can recover the downregulation of MLC phosphorylation that is induced by ROCK inhibition.

### Immunohistological analyses of nasal mucosal cell sheets

Cell–cell junctions were evaluated using immunofluorescence experiments and Western blotting to detect E-cadherin, claudin-1 and ZO-2 (Figs. 4C, S6A,B; full-length western blot in Fig. S7). Since cells were unable to form cell sheets under low Ca^2+^ conditions, immunohistochemistry experiments could not be performed for cell sheets cultured in 0.1 mM Ca^2+^. Each cell sheet was composed of several layers, so immunohistology was performed to evaluate the cell–cell junctions in each layer. Immunohistology detected E-cadherin in almost all cells under all cell culture conditions. However, poor cell–cell adhesions were observed in the basal layer of cell sheets cultivated in 1.6 mM Ca^2+^ (KCM-FBS) or 1.6 mM Ca^2+^ with 10 µM Y-27632. By contrast, tight adhesions between cells were observed when the medium contained 3.6 mM Ca^2+^ and 1 µM Y-27632, and E-cadherin was observed in the basal cell layer, suggesting that elevation of the Ca^2+^ level improved cell–cell adhesions in the basal layer directly or indirectly. We also confirmed the reproducibility of the P-MLC and E-cadherin expression regardless of the condition of the original tissue (*n* = 4, Fig. S4). TEM images also demonstrated gaps between cells in the basal layer when medium containing 1.6 mM Ca^2+^ or medium containing 1.6 mM Ca^2+^ and 10 µM Y-27632 was used but little or no gaps when medium containing 3.6 mM Ca^2+^ and 1 µM Y-27632 was used (Fig. S6C–E). Immunohistology showed that claudin-1 and ZO-2 were present at the periphery of each cell when medium containing 1.6 mM Ca^2+^ or medium containing 3.6 mM Ca^2+^ and 1 µM Y-27632 was used, but these proteins were rarely observed when medium containing 1.6 mM Ca^2+^ and 10 µM Y-27632 was utilized (Fig. 4C). Western blotting revealed no notable differences in the expressions of E-cadherin, claudin-1 and ZO-2 between the various culture conditions (Fig. S6B), and tight junctions were observed in TEM images (Fig. S6C–E). P-MLC was detected almost exclusively in the upper layer of the cell sheet when medium containing 1.6 mM Ca^2+^ or medium containing 1.6 mM Ca^2+^ and 10 µM Y-27632 was used. When the culture medium contained 3.6 mM Ca^2+^ and 1 µM Y-27632, P-MLC was present in all layers, suggesting that this combination of Ca^2+^ and Y-27632 promoted strong cell–cell adhesion within the basal layer. Expression of the proliferation marker Ki67 was similar between the various experimental conditions.

### Potential wound healing effects of cultured nasal epithelial cell sheets

**Figure 5:**
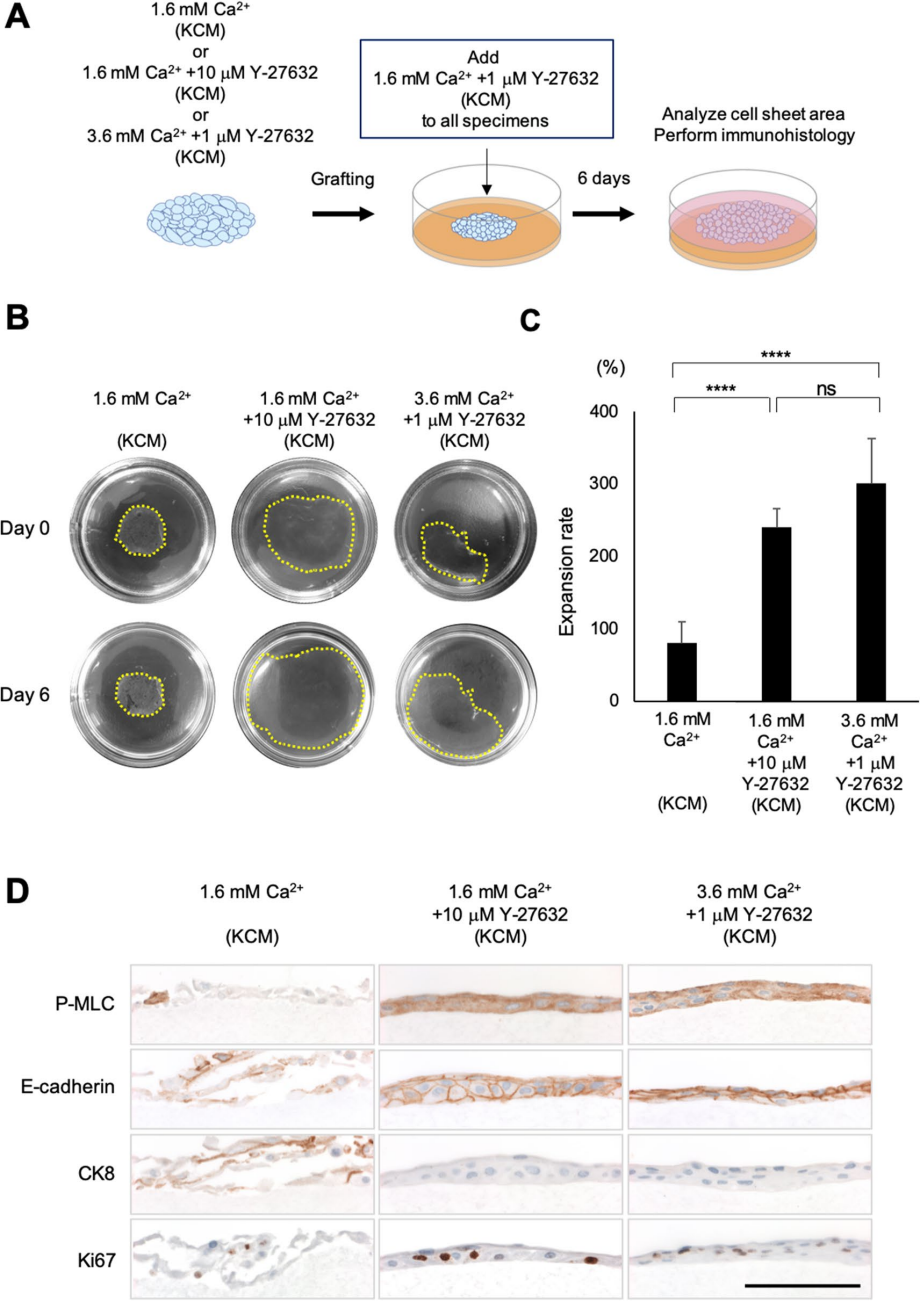
In vitro evaluation of the wound healing potential of cell sheets cultured in KCM containing 1.6 mM Ca^2+^, KCM containing 1.6 mM Ca^2+^  + 10 μM Y-27632, or KCM containing 3.6 mM Ca^2+^  + 1 μM Y-27632. (**A**) Schematic diagram of the in vitro cell sheet grafting assay. A harvested cell sheet was grafted onto a type I collagen gel and then cultivated for 6 days in KCM containing 1.6 mM Ca^2+^ and 1 μM Y-27632. (**B**) Representative images showing cell sheets before and 6 days after grafting onto type I collagen gels in 60-mm dishes. Yellow dotted line indicates the edge of each cell sheet. (**C**) Expansion rate 6 days after grafting onto the collagen gel. ****P < 0.0001; ns, not significant. (**D**) Immunohistological evaluation of the cell sheets at 6 days after grafting. Scale bar = 100 μm.

To analyze the wound healing effect, we performed a cell sheet grafting assay in vitro (Fig. 5A), in which a cell sheet adhered to and migrated on a collagen gel (Fig. 5B). The expansion rate at 6 days after grafting was significantly higher for sheets fabricated in 1.6 mM Ca^2+^ and 10 µM Y-27632 (240 ± 26%, *n* = 6) or 3.6 mM Ca^2+^ and 1 µM Y-27632 (301 ± 62%, *n* = 6) than for sheets fabricated in 1.6 mM Ca^2+^ (81 ± 29%, *n* = 8, *P* < 0.0001; Fig. 5C). These findings imply that cell sheet culture in the presence of a ROCKi would increase the potential wound healing effects of nasal epithelial cell sheets.

Colony forming assays (CFAs) using 2000 cells from each cell sheet were also performed to assess proliferation potential. Colonies were formed by cells obtained from sheets fabricated under all three culture conditions (Fig. S8A), but the colony forming efficiency was significantly higher for culture medium containing 1.6 mM Ca^2+^ and 10 µM Y-27632 (3.6 ± 1.6%, *n* = 6, *P* < 0.01) or 3.6 mM Ca^2+^ and 1 µM Y-27632 (1.9 ± 0.9%, *n* = 6, *P* < 0.05) than for culture medium containing 1.6 mM Ca^2+^ (0.5 ± 0.4%, *n* = 6; Fig. S8B).

### Immunohistological analyses of cell sheets after grafting onto a collagen gel

Although cell sheets fabricated in 1.6 mM Ca^2+^ (normal KCM) showed maintenance of adhesion in macroscopic images (Fig. 5B), the detachment of some cells from the collagen gel was observed in microscopic images (Fig. 5D). Furthermore, P-MLC was rarely detected in cell sheets fabricated in 1.6 mM Ca^2+^, and although CK8 was evident, Ki67 was poorly expressed (Fig. 5D). These results suggest that cell sheets produced using KCM containing 1.6 mM Ca^2+^ underwent differentiation rather than proliferation after grafting onto collagen. By contrast, grafted cell sheets that had been fabricated in the presence of Y-27632 exhibited extensive expression of E-cadherin and P-MLC, slight expression of CK8 and notable expression of Ki67. These findings imply that cell sheets fabricated in the presence of a ROCKi underwent proliferation after grafting, which would be favorable for wound healing.

## Discussion

**Figure 6:**
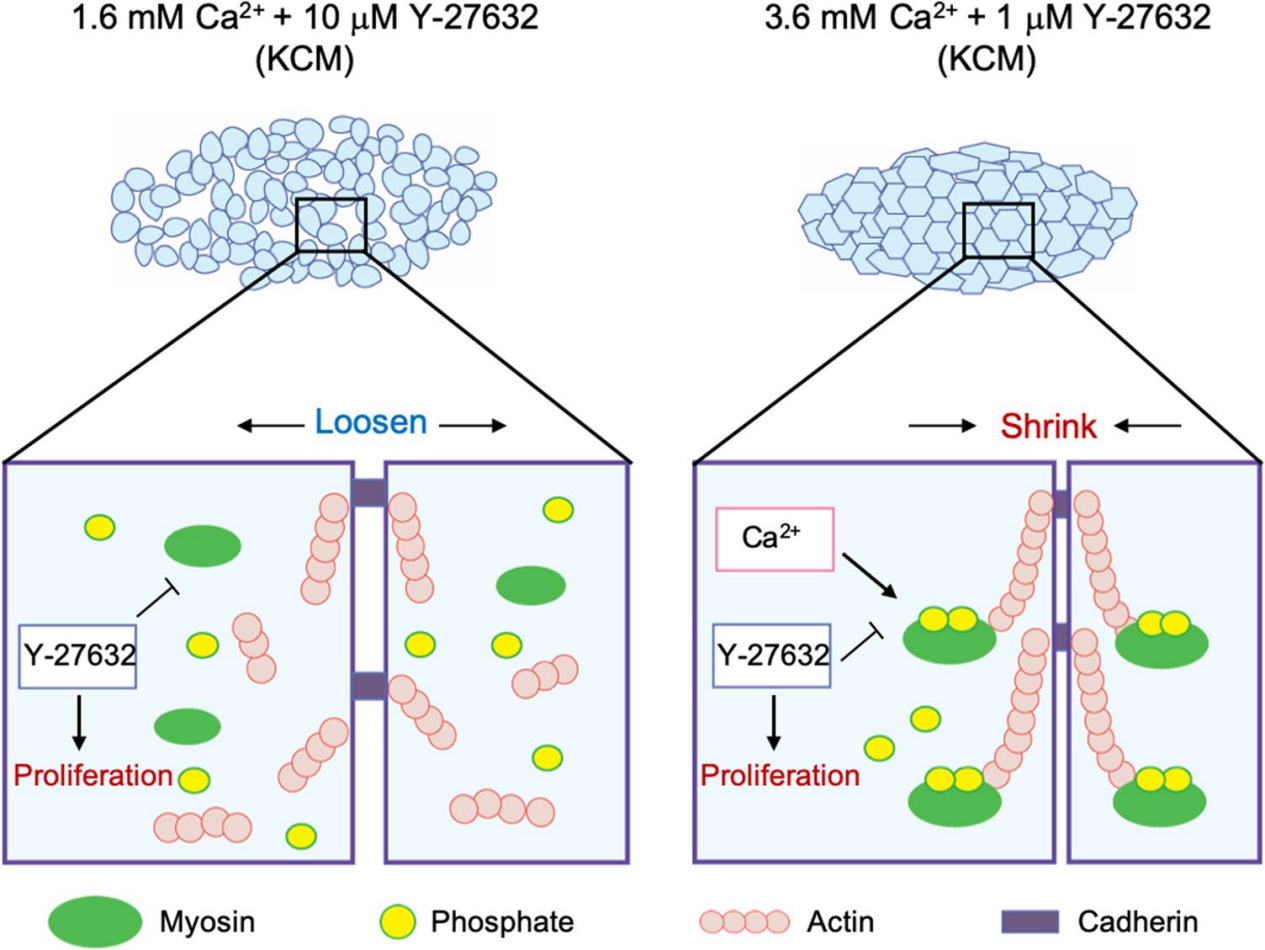
Schematic diagram summarizing the effects of ROCK inhibition and Ca^2+^ on cell sheet cultures. (**A**) In KCM containing 1.6 mM Ca^2+^ and 10 μM Y-27632, inhibition of ROCK promotes cell proliferation but dephosphorylates MLC to inhibit myosin–actin interactions, resulting in a loose cell sheet that contains defects after harvesting. (**B**) In KCM containing 3.6 mM Ca^2+^ and 1 μM Y-27632, the elevation of Ca^2+^ recovers MLC phosphorylation in the presence of the ROCK inhibitor, resulting in a cell sheet that shrinks on harvesting without developing defects.

The present study examined the use of Y-27632 (a ROCKi) and different concentrations of extracellular Ca^2+^ to optimize both explant culture and cell sheet culture. Furthermore, evaluation of the expressions of P-MLC and PP-MLC under different experimental conditions provided insights into the role of phosphorylated MLC in cell proliferation and differentiation during culture (Fig. 6). Our findings have enabled us to improve the fabrication of human nasal epithelial cell sheets and produce constructs that do not collapse after harvesting and have high wound healing potential in vitro.

Nasal mucosal tissue is a thin pseudostratified columnar epithelium^31^. Although the dispase dissociation technique is often used in the primary culture of skin grafts or oral mucosal cell sheets^32,33^, it is not considered suitable for the cultivation of nasal mucosal epithelial cells^34^. As we agree with the findings of this latter report, we used primary explant culture as a cell expansion stage (Fig. 1A). Since low-Ca^2+^ medium is widely used to expand epithelial cells^26^, we examined the use of KGM (0.1 mM Ca^2+^) as a low-Ca^2+^ medium for explant culture. However, the tissue adhesion rate was notably lower in medium containing 0.1 mM Ca^2+^ than in medium containing 1.1 mM Ca^2+^ (Fig. 1B). As the adhesion of fibroblasts requires an extracellular Ca^2+^ concentration of at least 0.5 mM^26,35^, we suggest that increasing the level of Ca^2+^ in the medium facilitated the initial adhesion of the explant culture. This proposal is supported by our histological analysis of the explant culture, which demonstrated a connective tissue layer beneath the epithelial layer as well as the occurrence of fibroblast adhesion before epithelial cell migration (Fig. 2B). Therefore, we believe that connective tissue is key to adhesion in explant culture and that Ca^2+^ is important for the development of adhesion. Bivalent metal ions such as Mg^2+^ and Mn^2+^ are known promoters of cell adhesion via integrin^36^. Since our optimization of explant culture based on ROCK inhibition and Ca^2+^ level allowed us to collect a sufficient number of cells for cell sheet fabrication, we did not examine the roles of other bivalent ions; thus, it is likely that there is further room for improvement of the explant culture technique. Epithelial mesenchymal transition (EMT) is understood to involve multiple steps of transition between epithelial and mesenchymal tissue, and these are now referred to as partial EMT^37,38^. In our experiments, CK8, a marker of differentiated epithelium, was not expressed in proliferating/migrating cells, whereas the epithelial marker E-cadherin and the mesenchymal marker vimentin were expressed in migrating cells (Fig. 2B). The above results suggest that partial EMT occurred in the explant culture.

Although some cell types have been successfully cultured in the absence of serum^39^, serums are still considered essential supplements for cell cultivation. Unexpectedly, nasal cells reached only 15% confluence in a 60-mm dish at day 13 when cultured in FBS-containing medium (normal KCM, Fig. 1C), whereas substantially higher confluence was achieved when human serum was used. Since improving the expansion of epithelial cells in medium containing γ-irradiated FBS would facilitate future preclinical and clinical research, we examined the effects of 10 μM Y-27632, a ROCKi that is known to enhance cell proliferation^17^. As expected, Y-27632 significantly promoted cell proliferation (Fig. 1C,D). Interestingly, proliferating epithelial cells mainly existed at the edge of the explant culture (Fig. 2B), indicating that cell behavior at the migrating edge plays an important role in cell expansion. When cultured in normal KCM (1.6 mM Ca^2+^), cells in the migrating edge exhibited partial backward movements (Movie 1) similar to those reported in our previous study^40^, and P-MLC and stress fibers were detected at the cell–cell interfaces (Fig. 2D,E), implying that P-MLC and actin may operate to connect migrating cells to each other, as suggested by a prior investigation using keratinocytes^41^. By contrast, P-MLC was not strongly detected, and actin depolymerization occurred at the cell–cell interfaces when culture was carried out in medium containing 1.6 mM Ca^2+^ and 10 µM Y-27632, implying weaker connections between migrating cells; this may have contributed to the enhancement of cell proliferation and some of the cells migrating individually (Fig. 2F). Based on these results, we suggest that P-MLC at the cell periphery prevents cell proliferation (Fig. 2D,G), consistent with a previous report that cell proliferation is induced by the mosaic loss of myosin and 50–75% of MLC^11^.

The purpose of cell sheet culture is to connect cells to each other so that all the cells can be detached as an intact construct. Claudin-1 and ZO-2 were expressed in the upper layer of the cell sheet under all experimental conditions (Fig. S6), indicating the presence of tight junctions^42,43^. Although adherens junctions were observed in the basal layer by TEM when normal KCM (1.6 mM Ca^2+^) was used (Fig. S6), the basal layer was found to contain many intercellular gaps (Fig. 4C). Therefore, we speculated that the addition of a ROCKi to promote continuous proliferation would prevent the formation of basal layer gaps. However, although we confirmed that Y-27632 downregulated P-MLC (Fig. 4A,B), the resulting cell sheets were structurally loose and still contained some defects (Figs. 3B, S4). Based on these results, we concluded that the prevention of intercellular defects in the basal layer required both cell proliferation and MLC activation, which would necessitate the recovery of MLC phosphorylation in the presence of the ROCKi. In cardiovascular smooth muscle, phosphorylation of MLC is also regulated by Ca^2+^ through a different pathway to that recruited by ROCK^44^. In epithelia, non-muscle P-MLC enhances the accumulation of E-cadherin-related proteins^45^. Therefore, we examined whether increasing the level of Ca^2+^ in Y-27632-containing medium (3.6 mM Ca^2+^ and 1 µM Y-27632) would recover MLC phosphorylation (and thus activation) and promote cell–cell adhesion. When medium containing 3.6 mM Ca^2+^ and 1 µM Y-27632 was used, P-MLC and E-cadherin were expressed at the cell–cell interfaces of all layers (Fig. 4A–C), and the cell sheet could be harvested without collapse and shrinkage (Fig. 3B). We propose that P-MLC participates in the shrinkage behavior of nasal mucosal cell sheets, which has also been observed in keratinocyte sheets^40,46^. These results suggest that increasing the Ca^2+^ concentration in the culture medium recovered MLC phosphorylation and thereby promoted the maturation of adherens junctions (Figs. 3B, 4A–C). Overall, our findings demonstrate that the combination of a ROCKi with Ca^2+^ induces MLC phosphorylation/activation in all layers of the cell sheet, thereby promoting cell–cell adhesion and facilitating the harvesting of an intact cell sheet that does not collapse.

Finally, we used an in vitro cell sheet grafting assay and CFA to analyze the wound healing ability of the optimized cell sheet (Figs. 5, S8). We previously established an experimental model to evaluate grafted cell sheets in vitro and used this model to confirm the behavior of normal human epidermal keratinocyte cell sheets^40^. Therefore, we used this model system to analyze the behavior of cell sheets fabricated under three culture conditions (Fig. 5A). Cell sheets fabricated in KCM containing 1.6 mM Ca^2+^ and 10 µM Y-27632 or KCM containing 3.6 mM Ca^2+^ and 1 µM Y-27632 expanded nearly three-fold relative to their original size and approximately three-fold relative to the expansion of cell sheets made using medium containing 1.6 mM Ca^2+^ (Fig. 5B,C). Ki67 was rarely detected in cell sheets before grafting, suggesting that proliferation had ceased because of confluence (Fig. 4C). However, cell sheets expressed Ki67 after grafting, indicating that they had proliferated on the collagen gel (Fig. 5D). The CFA results supported the findings of the cell sheet grafting assays (Fig. S8A, B). Although the wound healing potential in vitro was improved by the combination of a ROCKi with Ca^2+^, it should be emphasized that the environment in which the cell sheet was grafted was different to that in vivo. Nevertheless, since maintaining proliferative potential is important for effective therapy^47–49^, we consider our graft to have potential for use in the clinical setting.

There are two main limitations to this study. First, inhibitors of ROCK affect not only myosin regulation but also gene expression^50,51^, but we did not perform gene expression assays. It will be important to investigate the expressions of genes at each step of the cell culture process (original tissue, explant culture, cell sheet and grafted cell sheet) to better understand the changes that occur and how these are influenced by inhibition of ROCK or variations in Ca^2+^ level, since this knowledge may facilitate further optimization of cell sheets suitable for grafting. Second, we did not examine the populations of various cell types in the cell sheet. Native nasal mucosal tissue contains different cell types, including fibroblasts, basal epithelial cells, goblet cells and ciliated cells, and these various cell types potentially influence the wound healing potential. Further work is needed to address these limitations.

Regenerative medicine using cultured cell grafts is an important research field in cell biology. Recent studies fabricating similar cell grafts have utilized multiple cell culture steps including cell expansion and cell differentiation^52,53^. The fabrication of a cell graft that is fully compatible with the transplant site will require an optimal balance of several agents in the culture medium. Here, we demonstrate that a balanced combination of a ROCKi and Ca^2+^ can maintain MLC phosphorylation and optimize both explant culture and cell sheet culture using human nasal mucosal cells. Our study provides important new insights into the role of phosphorylated MLC in the behavior of cell clusters, and we anticipate that our findings will facilitate the fabrication of stable cell grafts for use in regenerative medicine.

## Materials and methods

### Surgical collection of human nasal mucosal tissue

All methods were performed in accordance with the Declaration of Helsinki and Ethical Guidelines for Medical and Health Research Involving Human Subjects in Japan. In addition, this study was approved by the Institutional Review Board of Jikei University, Tokyo, Japan. All volunteers, who were scheduled to undergo endoscopic sinus surgery, provided informed consent and were confirmed not to be infected with human immunodeficiency virus, syphilis, hepatitis B virus or hepatitis C virus. Nasal mucosal tissue was collected from the inferior turbinate.

### Culture media

KGM (Lonza, Basel, Switzerland) was used as the low-Ca^2+^ medium (0.1 mM Ca^2+^). Normal KCM containing 1.6 mM Ca^2+^ was prepared by mixing equal volumes of DMEM (Thermo Fisher Scientific, Waltham, MA, USA) and DMEM with Ham’s F-12 medium (Thermo Fisher Scientific). In some experiments, γ-irradiated FBS (Sigma-Aldrich, St. Louis, MO, USA) was added at a concentration of 10%. KCM was supplemented with 0.3 μM hydrocortisone (SAXIZON, Takeda Yakuhin Kogyo, Nihonbashi, Tokyo, Japan), 140.0 mU/mL insulin (Novo Nordisk, Bagsværd, Copenhagen, Denmark), 2.0 nM triiodothyronine (Sigma-Aldrich), 0.2 μM epidermal growth factor (Higeta-Shoyu, Nihonbashi, Tokyo, Japan), 1.0 nM cholera toxin (Wako Pure Chemical Industries, Dosyomachi, Osaka, Japan), 100 U/mL penicillin (Meiji Seika Pharma, Kyobashi, Tokyo, Japan), 69 μM streptomycin (Meiji Seika Pharma) and 0.4 μg/mL amphotericin B (Bristol-Myers Squibb, New York City, NY, USA). Depending on the experiment, Y-27632 (Wako Pure Chemical Industries) was added to obtain a final concentration of 10 μM, 2.5 μM, 1 μM or 100 nM, and CaCl_2_ was added to increase the Ca^2+^ concentration by 1 mM, 2 mM or 4 mM. In some experiments, KCM was supplemented with 5% autologous human serum (KCM-HS) instead of FBS. Human blood was collected from volunteers scheduled to undergo endoscopic sinus surgery (each supplied 70 mL), and serum was extracted according to our previous paper^30^.

### Explant culture

Nasal mucosal tissues underwent explant culture using a modification of our previously described method^30^. In short, sterilized mucosal tissues were cut into 1.5-mm cubes, placed in cell culture dishes (Primaria Dish, Corning, Corning, NY, USA) and incubated in 750 μL of medium at 37 °C with 5% CO_2_ (Fig. 1A). A further 1 mL of medium was added to each dish 1 h later, and another 2 mL of medium was added the following day. The medium was changed on days 6 and 10. Following 13 days of cultivation, cells were collected by trypsin–EDTA treatment and preserved with freezing medium (STEM-CELLBANKER, Nippon Zenyaku Kogyo, Asakamachi, Fukushima, Japan). In order to track migration behavior, cell outgrowth was monitored for 8 h on day 7 of the explant culture process using differential interference contrast microscopy (CytoWatcher, Atto, Motoasakusa, Tokyo, Japan).

### Cell sheet culture

Cells were thawed, seeded on temperature-responsive cell culture dishes (CellSeed, Ōme, Tokyo, Japan) at a density of 1.0 × 10^5^ cells/cm^2^ and cultured for 7 days at 37 °C (Fig. 3A). The medium was changed on days 3 and 5. After 7 days of culture, the cells were detached as a cell sheet by reducing the temperature to 20 °C for 40 min.

### Immunofluorescence analysis of cultured cells

Nasal mucosal tissues or nasal mucosal cells were cultured on a slide chamber (Thermo Fisher Scientific) and fixed in 100% ethanol or 4% paraformaldehyde (Wako Pure Chemical Industries). Sections were exposed to blocking buffer (Nacalai Tesque, Nijo Karasuma, Nakagyo-ku, Kyoto, Japan) and then incubated with phosphate-buffered saline (PBS) containing primary antibody (Table S1) at 4 °C overnight. After washing with PBS, the samples were incubated with Alexa Fluor Plus 488-conjugated secondary antibody (Table S1) for 1 h at room temperature. The nuclei were stained using 4′,6-diamidino-2-phenylindole (DAPI; Thermo Fisher Scientific), and F-actin was stained by phalloidin (Thermo Fisher Scientific) at room temperature for 1 h.

### Immunohistological analysis of nasal mucosal cell sheets

Immunohistology was performed according to methods described in our previous article^30^. Fabricated cell sheets were fixed in 4% paraformaldehyde (Wako Pure Chemical Industries), dehydrated, embedded in paraffin, sectioned (4 μm) and mounted on glass slides. The slides were de-paraffinized, and antigen retrieval was performed by autoclaving with citrate buffer (Histo-VT One, Nacalai Tesque). Peroxidase blocking (Dako) and protein blocking (Nacalai Tesque) were carried out to prevent non-specific reactions. The slides were then incubated overnight at 4 °C in PBS containing primary antibody (Table S2). After washing with PBS, the slides were incubated with horseradish peroxidase (HRP)-conjugated secondary antibodies (EnVision Detection System, Peroxidase/DAB, Rabbit/Mouse, HRP; K5007; Dako) at room temperature for 1 h. The sections were washed again and treated with 3,3′-diaminobenzidine (K5007; Dako) to visualize the HRP. Nuclei were stained by hematoxylin. De-paraffinized sections were also stained with hematoxylin and eosin (HE) for histological evaluation.

### Western blot analysis

Western blotting was performed using a modification of the method described in our previous study^54^. An entire cell sheet was solubilized in 855 μL Laemmli sample buffer (Bio-Rad, Hercules, CA, USA) containing 45 μL 2-mercaptoethanol and 9 μL proteinase/phosphatase inhibitor (Cell Signaling Technology). Samples (5 μL) were loaded onto a polyacrylamide gel (Thermo Fisher Scientific), and electrophoresis was performed. After transfer to a nitrocellulose membrane using a gel transfer device (Thermo Fisher Scientific), the membrane was blocked with 5% skim milk (Cell Signaling Technology) in Tris-buffered saline/Tween 20 (TBS-T) for 1 h at room temperature. The membrane was incubated with primary antibodies (Table S3) diluted in 2.5% blocking buffer at 4 °C overnight. After being washed with TBS-T, the membrane was incubated with secondary antibody (Table S3) for 1 h at room temperature. The membrane was washed with TBS-T and treated with luminol (ECL Prime, GE Healthcare, Chicago, IL, USA) for 5 min to detect the HRP. Photographs were taken using a chemiluminescence imager (LAS 4000; GE Healthcare).

### In vitro wound healing assay

Wound healing assays (Fig. 4C) were performed in vitro using a modification of a method described previously^40^. Briefly, a nasal cell sheet was grafted onto type I collagen gel in a 60-mm dish, and silicone that was covered by nylon mesh (Sansyo, Kandasakumacho, Tokyo, Japan) was used as a weight to promote adhesion of the cell sheet to the collagen gel. The weight was removed after incubation at 37 °C for 2–4 h. On the following day, 2 mL of KCM plus 1 μM Y-27632 was added to the culture dish. Adherent cell sheets were cultured for 6 days, with the medium changed on days 3 and 5.

### Colony-forming assay (CFA)

Colony-forming efficiency was calculated according to a previously reported method^55^. A CFA was used to measure the proliferative potential of cell sheets cultured under various experimental conditions. Harvested cell sheets were treated with trypsin–EDTA at 37 °C to obtain a suspension of dissociated cells. Two thousand cells were seeded in a 60-mm dish containing KCM and 1 μM Y-27632. The medium was changed on days 5 and 10. After 14 days, the colonies were fixed by 4% paraformaldehyde and stained with crystal violet solution (Merck, Darmstadt, Germany).

### Transmission electron microscopy (TEM)

TEM was carried out using a modification of a method reported previously^56^. Briefly, harvested cell sheets were fixed in 2.0% glutaraldehyde in 0.1 M phosphate buffer, treated with 1% osmium tetroxide in 0.1 M phosphate buffer, dehydrated in ethanol, immersed in absolute propylene oxide and embedded in Epok 812 (Okensyoji, Ginza, Tokyo, Japan). We confirmed the trimming regions using toluidine blue-stained sections. Ultrathin sections (approximately 60 nm) were prepared, mounted on grids, stained with uranyl acetate and lead citrate, and observed using TEM (H-7500, Hitachi, Marunouchi, Tokyo, Japan) at 80 kV.

### Statistical analysis

Prism 7.0 (GraphPad, San Diego, CA, USA) was used for the statistical analysis. Data are presented as the mean ± standard error of the mean (SEM). Comparisons between two groups were made using Student’s *t*-test. Comparisons between three groups were made using one-way analysis of variance and the Bonferroni or Tukey–Kramer multiple comparisons test as the post-hoc test. *P*-values < 0.05 were considered statistically significant.

## Supplementary information

Supplementary Movie 1.

Supplementary Movie 2.

Supplementary Information.

## References

[1] Green H (1978). Cyclic AMP in relation to proliferation of the epidermal cell: a new view. Cell.

[2] Rheinwald JG, Green H (1977). Epidermal growth factor and the multiplication of cultured human epidermal keratinocytes. Nature.

[3] Takagi R (2015). How to prevent contamination with *Candida albicans* during the fabrication of transplantable oral mucosal epithelial cell sheets. Regen. Ther..

[4] Formanek M (1996). Optimized growth medium for primary culture of human oral keratinocytes. Int. J. Oral Maxillofac. Surg..

[5] Nishida K (2004). Corneal reconstruction with tissue-engineered cell sheets composed of autologous oral mucosal epithelium. N. Engl. J. Med..

[6] Ohki T (2012). Prevention of esophageal stricture after endoscopic submucosal dissection using tissue-engineered cell sheets. Gastroenterology.

[7] Jonas E (2016). Transplantation of tissue-engineered cell sheets for stricture prevention after endoscopic submucosal dissection of the oesophagus. United Eur. Gastroenterol. J.

[8] Yamaguchi N (2017). Oral epithelial cell sheets engraftment for esophageal strictures after endoscopic submucosal dissection of squamous cell carcinoma and airplane transportation. Sci. Rep..

[9] Yamamoto K (2017). Middle ear mucosal regeneration by tissue-engineered cell sheet transplantation. NPJ. Regen. Med..

[10] Vicente-Manzanares M, Ma X, Adelstein RS, Horwitz AR (2009). Non-muscle myosin II takes centre stage in cell adhesion and migration. Nat. Rev. Mol. Cell Biol..

[11] Nguyen-Ngoc KV (2017). Mosaic loss of non-muscle myosin IIA and IIB is sufficient to induce mammary epithelial proliferation. J. Cell Sci..

[12] Boraas LC, Pineda ET, Ahsan T (2018). Actin and myosin II modulate differentiation of pluripotent stem cells. PLoS ONE.

[13] Riento K, Ridley AJ (2003). Rocks: multifunctional kinases in cell behaviour. Nat. Rev. Mol. Cell Biol..

[14] Wilkinson S, Paterson HF, Marshall CJ (2005). Cdc42-MRCK and Rho-ROCK signalling cooperate in myosin phosphorylation and cell invasion. Nat. Cell Biol..

[15] Schwartz M (2004). Rho signalling at a glance. J. Cell Sci..

[16] Uehata M (1997). Calcium sensitization of smooth muscle mediated by a Rho-associated protein kinase in hypertension. Nature.

[17] Watanabe K (2007). A ROCK inhibitor permits survival of dissociated human embryonic stem cells. Nat. Biotechnol..

[18] Aslanova A (2015). A chemically defined culture medium containing Rho kinase inhibitor Y-27632 for the fabrication of stratified squamous epithelial cell grafts. Biochem. Biophys. Res. Commun..

[19] Kinoshita S (2018). Injection of cultured cells with a ROCK inhibitor for bullous keratopathy. N. Engl. J. Med..

[20] Scholey JM, Taylor KA, Kendrick-Jones J (1980). Regulation of non-muscle myosin assembly by calmodulin-dependent light chain kinase. Nature.

[21] Adelstein RS, Conti MA, Pato MD (1980). Regulation of myosin light chain kinase by reversible phosphorylation and calcium-calmodulin. Ann. N. Y. Acad. Sci..

[22] Ma XL, Liu HQ (2011). Effect of calcium on the proliferation and differentiation of murine corneal epithelial cells in vitro. Int. J. Ophthalmol..

[23] Takeichi M (1977). Functional correlation between cell adhesive properties and some cell surface proteins. J. Cell Biol..

[24] D'Souza SJ, Pajak A, Balazsi K, Dagnino L (2001). Ca^2+^ and BMP-6 signaling regulate E2F during epidermal keratinocyte differentiation. J. Biol. Chem..

[25] Hiraki A (2002). Calcium induces differentiation of primary human salivary acinar cells. J. Cell Physiol..

[26] Hennings H (1980). Calcium regulation of growth and differentiation of mouse epidermal cells in culture. Cell.

[27] Peehl DM, Stamey TA (1986). Serum-free growth of adult human prostatic epithelial cells. In Vitro Cell Dev. Biol..

[28] Zhang C (2018). Long-term *in vitro* expansion of epithelial stem cells enabled by pharmacological inhibition of PAK1-ROCK-Myosin II and TGF-beta signaling. Cell Rep..

[29] Hama T (2017). Autologous human nasal epithelial cell sheet using temperature-responsive culture insert for transplantation after middle ear surgery. J. Tissue Eng. Regen. Med..

[30] Kasai Y (2019). Analysis of human nasal mucosal cell sheets fabricated using transported tissue and blood specimens. Regen. Ther..

[31] Jafek BW (1983). Ultrastructure of human nasal mucosa. Laryngoscope.

[32] Kasai Y (2020). A stable protocol for the fabrication of transplantable human oral mucosal epithelial cell sheets for clinical application. Regen. Ther..

[33] Ueda M (1995). Formation of epithelial sheets by serially cultivated human mucosal cells and their applications as a graft material. Nagoya J. Med. Sci..

[34] Noruddin NAA, Saim AB, Chua KH, Idrus R (2007). Human nasal turbinates as a viable source of respiratory epithelial cells using co-culture system versus dispase dissociation technique. The Laryngoscope.

[35] Boynton AL, Whitfield JF, Isaacs RJ, Tremblay R (1977). The control of human WI-38 cell proliferation by extracellular calcium and its elimination by SV-40 virus-induced proliferative transformation. J. Cell Physiol..

[36] Zhang K, Chen J (2012). The regulation of integrin function by divalent cations. Cell Adhes. Migr..

[37] Grigore AD (2016). Tumor budding: The name is EMT. Partial EMT. J. Clin. Med..

[38] Savagner P (2010). The epithelial-mesenchymal transition (EMT) phenomenon. Ann. Oncol.

[39] Gottipamula S, Muttigi MS, Kolkundkar U, Seetharam RN (2013). Serum-free media for the production of human mesenchymal stromal cells: a review. Cell Prolif..

[40] Kasai Y (2017). Cellular events and behaviors after grafting of stratified squamous epithelial cell sheet onto a hydrated collagen gel. FEBS. Open Bio.

[41] Zhang J (2005). Actin at cell–cell junctions is composed of two dynamic and functional populations. J. Cell Sci..

[42] Balda MS, Matter K (2008). Tight junctions at a glance. J. Cell Sci..

[43] Heinemann U, Schuetz A (2019). Structural features of tight-junction proteins. Int. J. Mol. Sci..

[44] Shimokawa H, Rashid M (2007). Development of Rho-kinase inhibitors for cardiovascular medicine. Trends Pharmacol. Sci..

[45] Gomez GA, McLachlan RW, Yap AS (2011). Productive tension: force-sensing and homeostasis of cell–cell junctions. Trends Cell Biol..

[46] Green H, Kehinde O, Thomas J (1979). Growth of cultured human epidermal cells into multiple epithelia suitable for grafting. Proc. Natl. Acad. Sci. USA.

[47] Rama P (2010). Limbal stem-cell therapy and long-term corneal regeneration. N. Engl. J. Med..

[48] Butler CR (2016). Rapid expansion of human epithelial stem cells suitable for airway tissue engineering. Am. J. Respir. Crit. Care Med..

[49] Islam R (2017). Tissue harvesting site and culture medium affect attachment, growth, and phenotype of ex vivo expanded oral mucosal epithelial cells. Sci. Rep..

[50] Piltti J (2017). Rho-kinase inhibitor Y-27632 and hypoxia synergistically enhance chondrocytic phenotype and modify S100 protein profiles in human chondrosarcoma cells. Sci. Rep..

[51] Maldonado M, Luu RJ, Ramos ME, Nam J (2016). ROCK inhibitor primes human induced pluripotent stem cells to selectively differentiate towards mesendodermal lineage via epithelial-mesenchymal transition-like modulation. Stem Cell Res..

[52] Konishi S (2016). Directed induction of functional multi-ciliated cells in proximal airway epithelial spheroids from human pluripotent stem cells. Stem Cell Rep..

[53] Camp JG (2017). Multilineage communication regulates human liver bud development from pluripotency. Nature.

[54] Kasai Y (2016). Brush biopsy of human oral mucosal epithelial cells as a quality control of the cell source for fabrication of transplantable epithelial cell sheets for regenerative medicine. Regen. Ther..

[55] Morino T (2018). Explant culture of oral mucosal epithelial cells for fabricating transplantable epithelial cell sheet. Regen. Ther..

[56] Tanaka K (2008). Apoptosis in the small intestine of neonatal rat using blue light-emitting diode devices and conventional halogen-quartz devices in phototherapy. Pediatr. Surg. Int..

