# Supplementary material for: ROCK inhibitor combined with Ca2+ controls the myosin II activation and optimizes human nasal epithelial cell sheets
Source: Sci Rep. 2020 Oct 8;10:16853. doi: 10.1038/s41598-020-73817-3 (PMC7544873; doi:10.1038/s41598-020-73817-3)
Supplement: Supplementary file 3 — Supplementary Information. [file 41598_2020_73817_MOESM3_ESM.pdf]

# Supplementary information

# ROCK inhibitor combined with $Ca^{2+}$ controls the myosin II activation and optimizes human nasal epithelial cell sheets

**Yoshiyuki Kasai[1*], Tsunetaro Morino[1], Eri Mori[1], Kazuhisa Yamamoto[1], Hiromi Kojima[1*]**

Affiliations: [1] Department of Otorhinolaryngology, Jikei University School of Medicine, Tokyo, Japan

* Corresponding authors: Yoshiyuki Kasai Ph.D. and Hiromi Kojima M.D. Ph.D.
Department of Otorhinolaryngology, Jikei University School of Medicine, 3-25-8 Nishi-Shinbashi, Minato-ku, Tokyo 105-8461, Japan
Institutional email address: ykasai@jikei.ac.jp, kojimah@jikei.ac.jp
Tel: +81-3-3433-1111, ext. 3601
Fax: +81-3-3578-9208

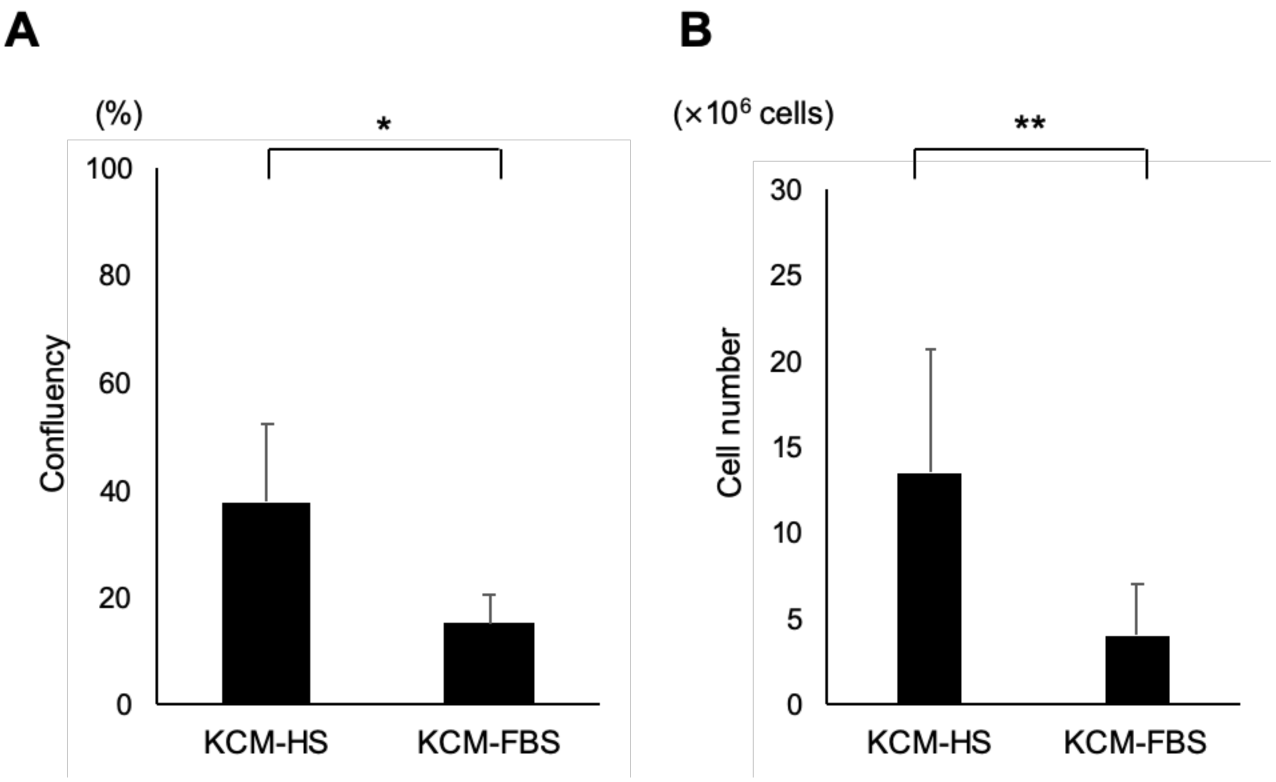


**Figure S1.** Cell expansion after explant culture. (A) Cell confluence in a 60-mm dish for cells cultured using normal KCM containing 5% human serum (HS; *n* = 5) or KCM containing 10% FBS (FBS; same as normal KCM *n* = 4). (B) Cell number counted from 8 dishes of cells cultured using KCM containing 5% human serum (HS) or normal KCM containing 10% FBS (FBS).

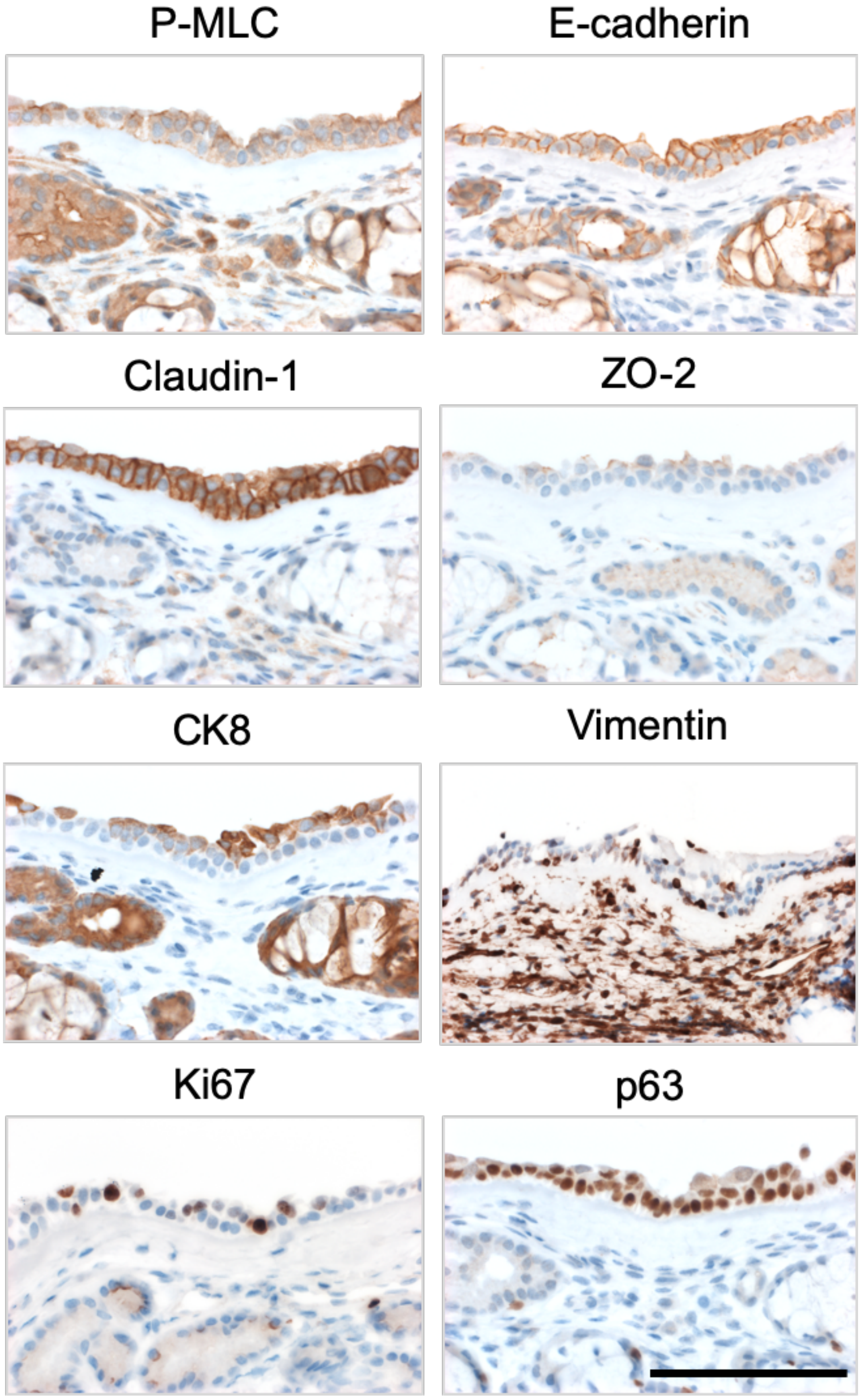


**Figure S2.** Immunohistological evaluation of human nasal mucosal tissue. Scale bar = 100 µm.

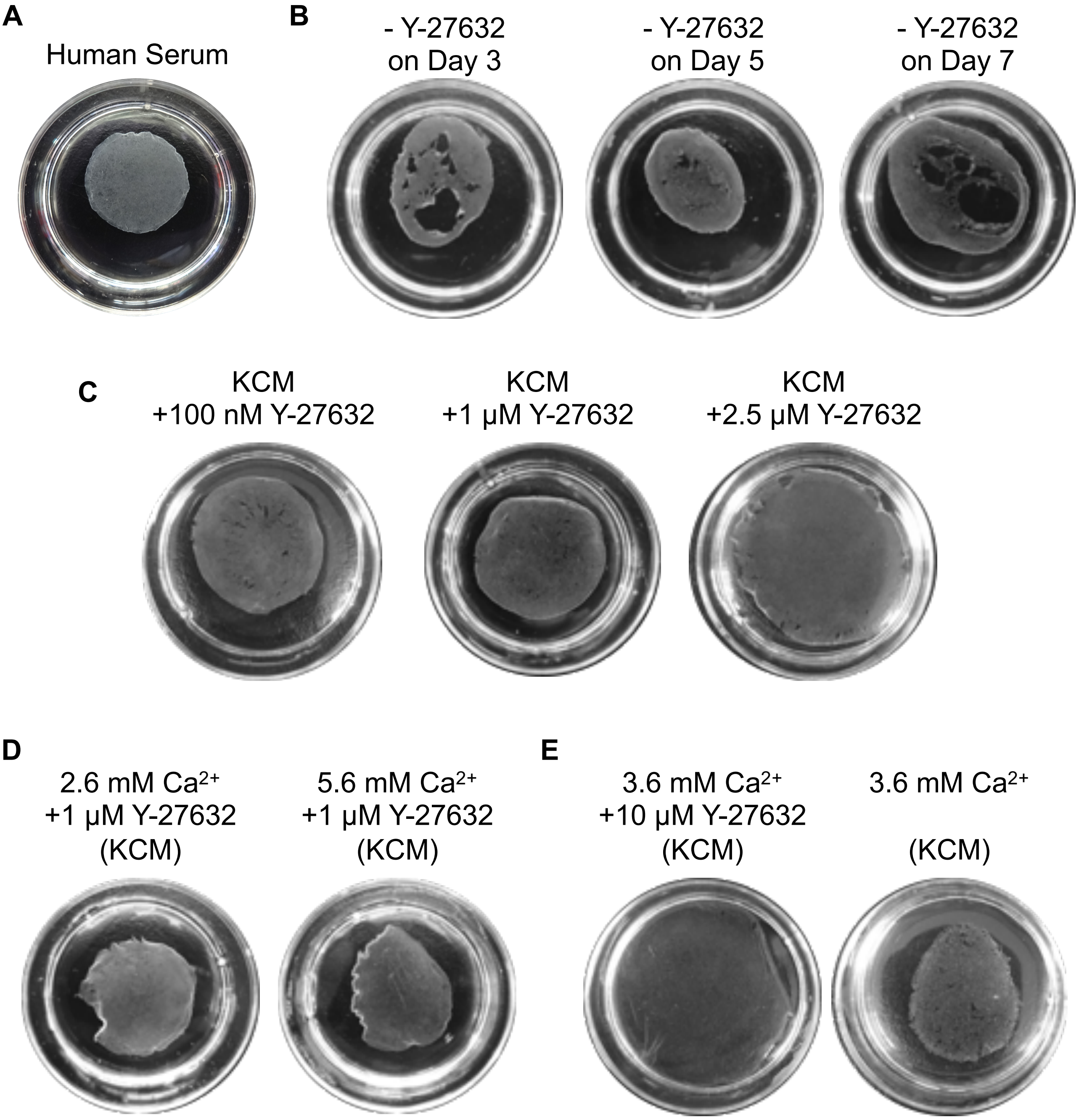


**Figure S3.** Representative images showing cell sheets cultured under various conditions. (A) Conventional method using autologous human serum. (B) Changing medium method that from KCM containing 1.6 mM $Ca^{2+}$ and 10 μM Y-27632 to KCM containing 1.6 mM $Ca^{2+}$ on day 0, 3, or 5. (C) Cell sheet cultured by different concentration of Y-27632. (D) Cell sheet cultured by KCM with 1 μM Y-27632 and different concentration of $Ca^{2+}$. (E) Cell sheet cultured by KCM containing 3.6 mM $Ca^{2+}$ + 10 μM Y-27632. (F) Cell sheet cultured by KCM containing 3.6 mM $Ca^{2+}$.

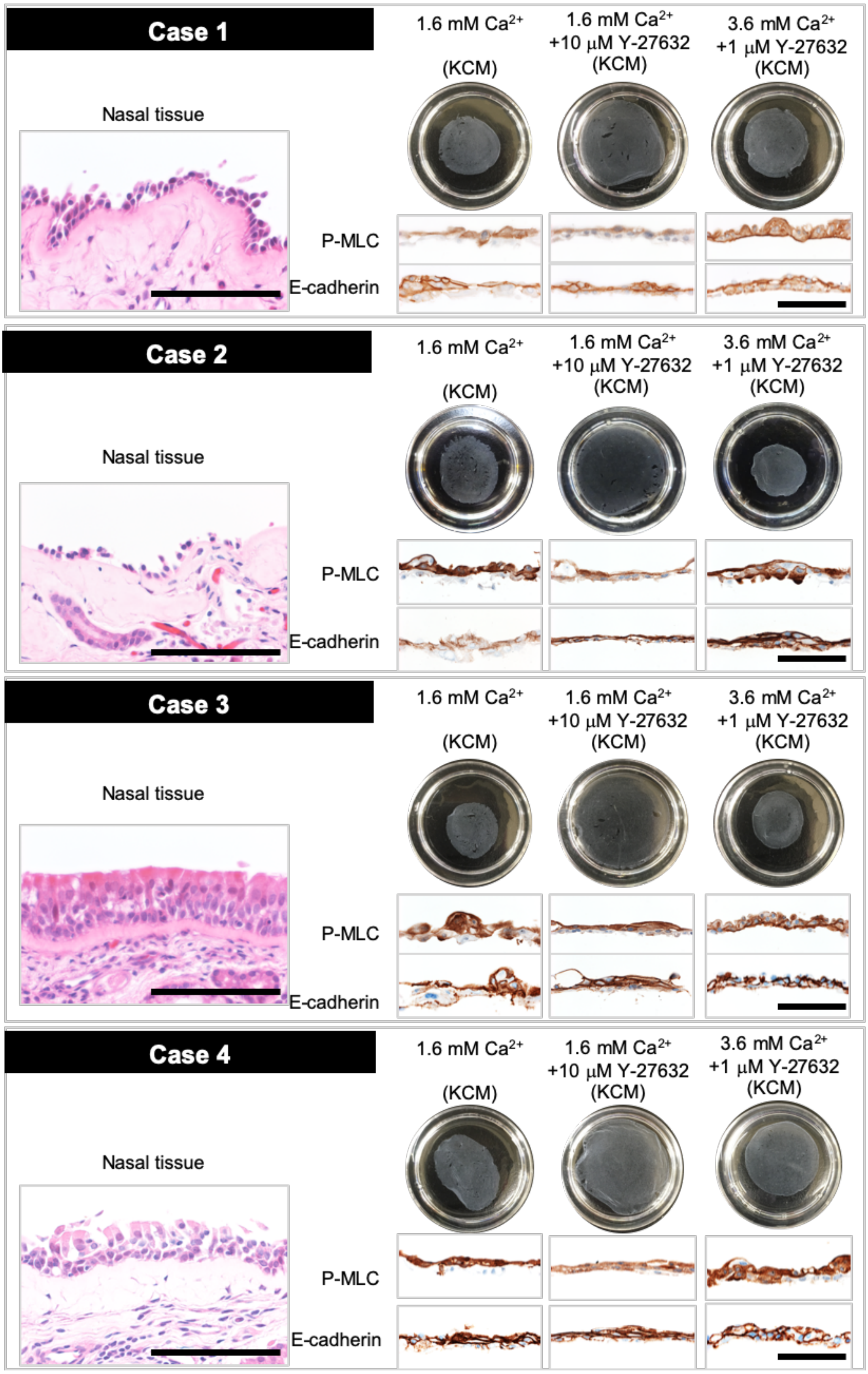


**Figure S4**. Reproducibility for shrinkage behavior and P-MLC and E-cadherin expression of a cell sheet independent on original tissue condition. Scale bar = 100 μm.

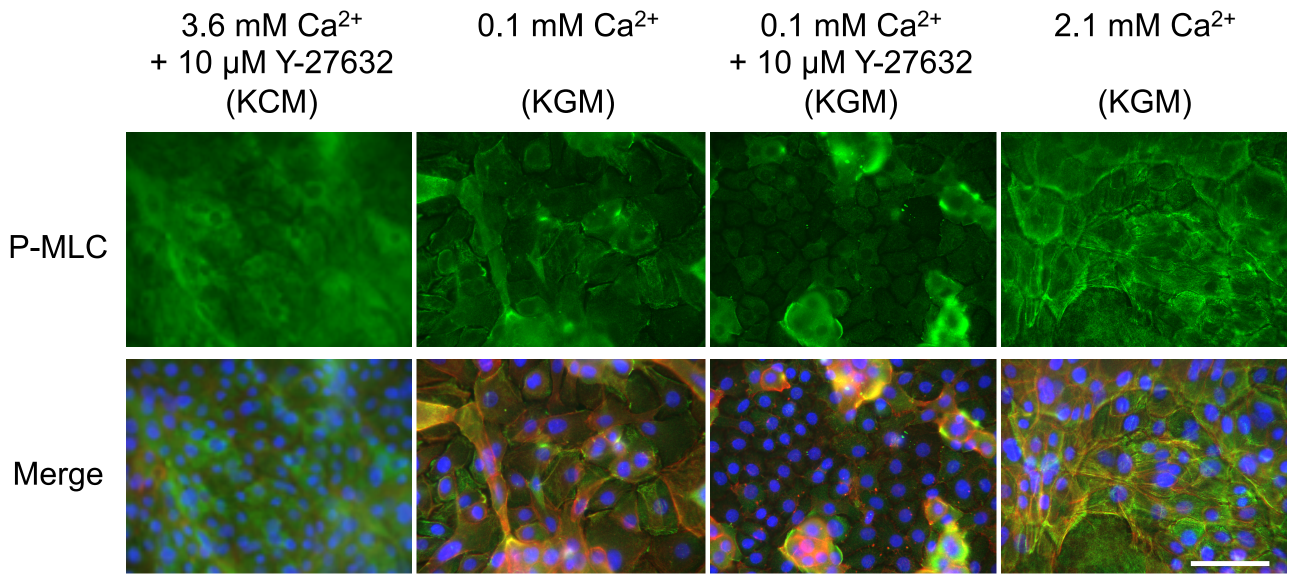


**Figure S5.** Immunofluorescence analysis of P-MLC expression. P-MLC (green), phalloidin (red), and DAPI (blue). Scale bar = 100 µm.

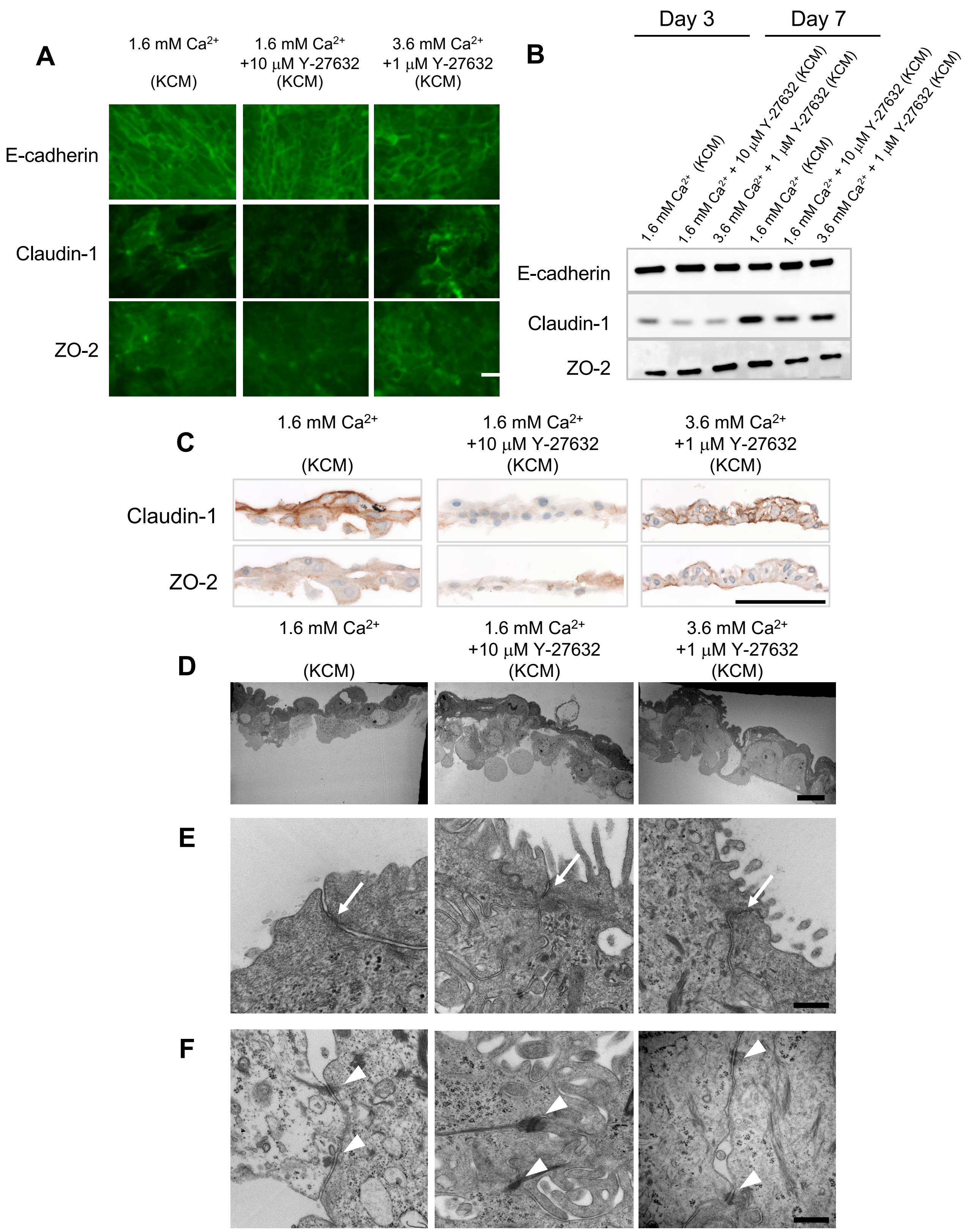


**Figure S6.** Analysis of cell-cell adhesion. (A) Immunofluorescence images showing the expressions of E-cadherin, claudin-1 and ZO-2 at day 7 of cell sheet culture. (B) Western blot analysis of the expressions of E-cadherin, claudin-1 and ZO-2 at day 3 and day 7 of cell sheet culture. The samples were the same as those used to obtain the data in Fig. 4C. (C) Immunohistological evaluation of the harvested cell sheet cultured in KCM containing 1.6 mM $Ca^{2+}$, KCM containing 1.6 mM $Ca^{2+}$ + 10 µM Y-27632, or KCM containing 3.6 mM $Ca^{2+}$ + 1 µM Y-27632. (D) TEM images of cell sheets

cultured cultured in KCM containing 1.6 mM $Ca^{2+}$, KCM containing 1.6 mM $Ca^{2+}$ + 10 μM Y-27632, or KCM containing 3.6 mM $Ca^{2+}$ + 1 μM Y-27632. (E) TEM images of the upper layer of each cell sheet. White arrows indicate tight junctions. (F) TEM images of the basal layer of each cell sheet. White arrowheads show adherens junctions. Scale bar = 100 μm (A, C), 20 μm (D), 500 nM (E, F).

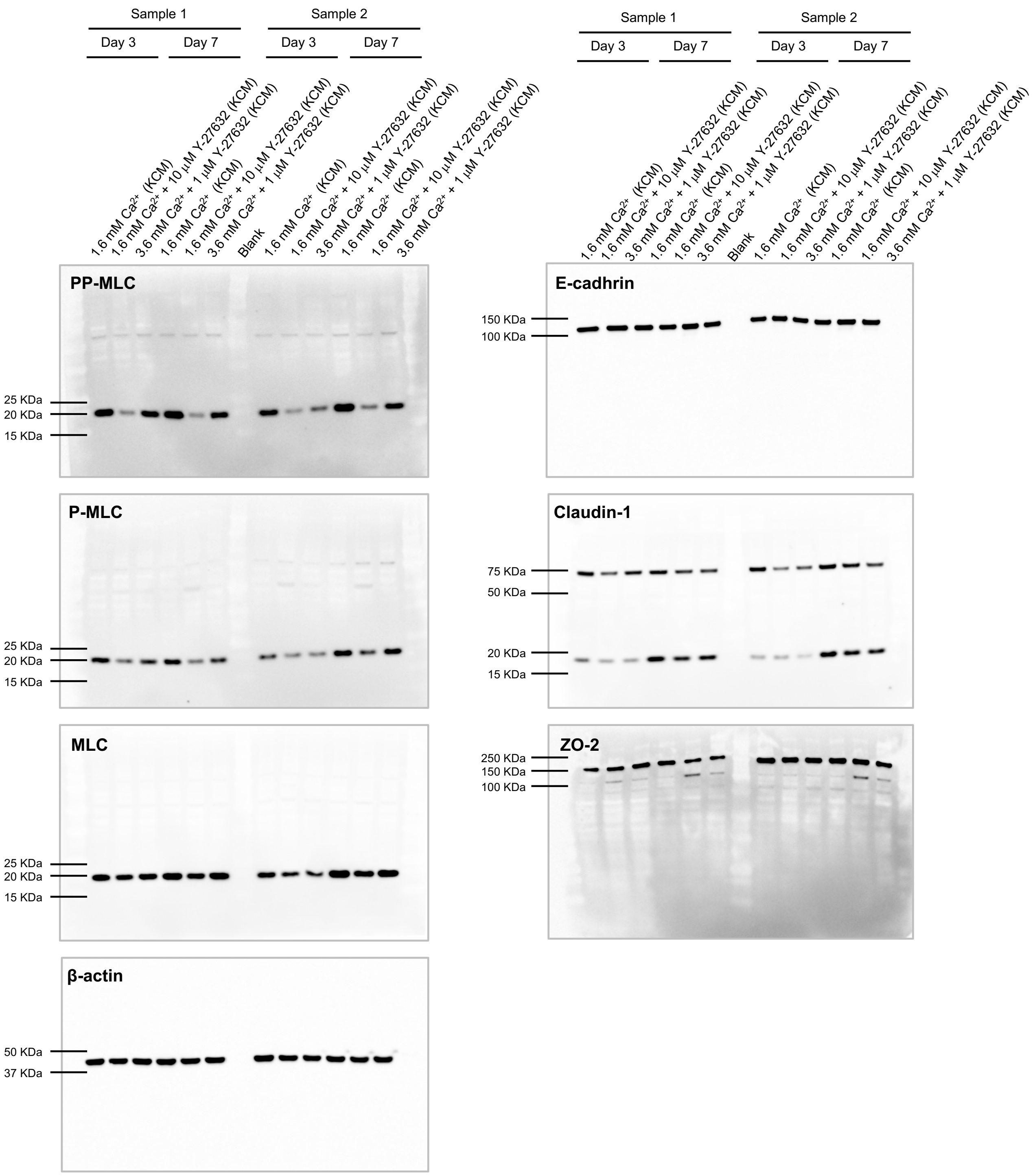


**Figure S7.** Full-length photograph of western blot shown in Figure 3D and Figure S4 ($n = 2$).

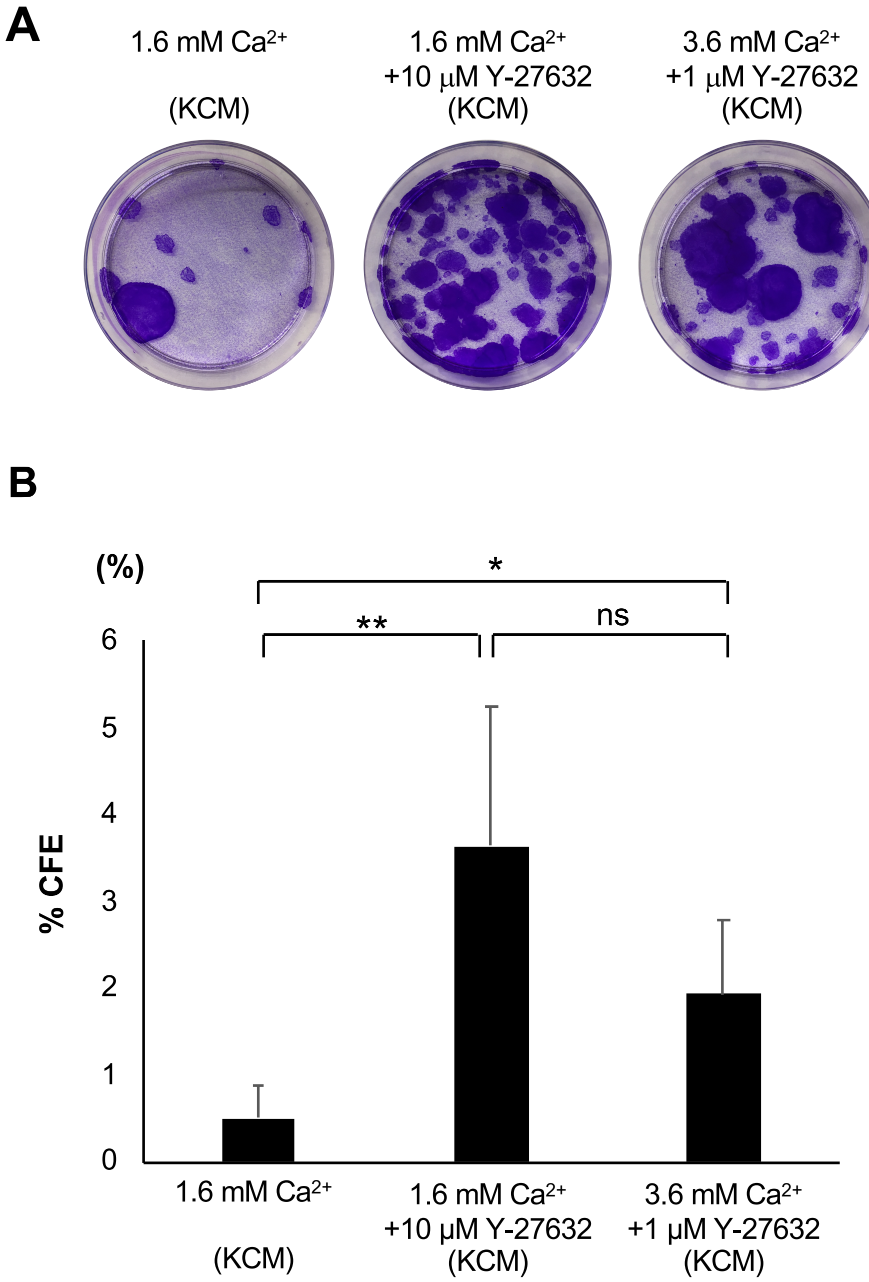


**Figure S8.** Evaluation of the colony forming efficacy (CFE) of the cell sheet cultured in KCM containing 1.6 mM $Ca^{2+}$, KCM containing 1.6 mM $Ca^{2+}$ + 10 μM Y-27632, or KCM containing 3.6 mM $Ca^{2+}$ + 1 μM Y-27632. (A) Representative images showing colony-forming assays for cell sheets. (B) Colony forming efficiency. Values are expressed as the mean ± SEM ($n$ = 6). *$P < 0.05$, **$P < 0.01$; ns, not significant.

Table S1: Antibodies used in Immunofluorescence analysis

| Antibodies | Cat.No. | Source | Dilution |
|---|---|---|---|
| Rabbit anti-human double-phospho-MLC polyclonal antibody (PP-MLC) | 3674 | Cell Signaling Technology, Danvers, MA, USA | 1:100 |
| Rabbit anti-human phospho-MLC polyclonal antibody (P-MLC) | 3671 | Cell Signaling Technology, Danvers, MA, USA | 1:100 |
| Rabbit anti-human MLC antibody (MLC)[D18E2] | 8505 | Cell Signaling Technology, Danvers, MA, USA | 1:100 |
| Rabbit anti-human claudin-1 monoclonal antibody [D5H1D] | 13255 | Cell Signaling Technology, Danvers, MA, USA | 1:100 |
| Rabbit anti-human zonula occludens-2 polyclonal antibody (ZO-2) | 2847 | Cell Signaling Technology, Danvers, MA, USA | 1:100 |
| Mouse anti-human E-cadherin monoclonal antibody [NCH-38] | M3612 | Dako, Carpinteria, CA, USA | 1:100 |
| Alexa Fluor Plus 488-conjugated secondary antibody: goat anti-rabbit IgG antibody | A32731 | Thermo Fisher Scientific, Waltham, MA, USA | 1:1000 |
| Alexa Fluor Plus 488-conjugated secondary antibody: goat anti-mouse IgG antibody | A32723 | Thermo Fisher Scientific, Waltham, MA, USA | 1:1000 |

Table S2: Antibodies used in Immunohistological analysis

| Antibodies | Cat.No. | Source | Dilution |
|---|---|---|---|
| Rabbit anti-human phospho-MLC polyclonal antibody (P-MLC) | 3671 | Cell Signaling Technology, Danvers, MA, USA | 1:100 |
| Rabbit anti-human claudin-1 monoclonal antibody [D5H1D] | 13255 | Cell Signaling Technology, Danvers, MA, USA | 1:100 |
| Rabbit anti-human zonula occludens-2 polyclonal antibody (ZO-2) | 2847 | Cell Signaling Technology, Danvers, MA, USA | 1:100 |
| Mouse anti-human E-cadherin monoclonal antibody [NCH-38] | M3612 | Dako, Carpinteria, CA, USA | 1:100 |
| Mouse anti-human Ki67 monoclonal antibody [MIB-1] | M7240 | Dako, Carpinteria, CA, USA | 1:100 |
| Mouse anti-human p63 monoclonal antibody [4A4] | ab735 | Abcam, Cambridge, UK | 1:100 |
| Mouse anti-human vimentin monoclonal antibody [V9] | MA5-11883 | Thermo Fisher Scientific, Waltham, MA, USA | 1:100 |
| Mouse anti-human cytokeratin-8 monoclonal antibody [M20] | sc-52324 | Santa Cruz Biotechnology, Dallas, CA, USA | 1:100 |

Table S3: Antibodies used in western blot analysis

| Antibodies | Cat.No. | Source | Molecular weight (kDa) | Dilution |
|---|---|---|---|---|
| Rabbit anti-human double-phospho-MLC polyclonal antibody (PP-MLC) | 3674 | Cell Signaling Technology, Danvers, MA, USA | 18 | 1:1000 |
| Rabbit anti-human phospho-MLC polyclonal antibody (P-MLC) | 3671 | Cell Signaling Technology, Danvers, MA, USA | 18 | 1:1000 |
| Rabbit anti-human MLC antibody (MLC)[D18E2] | 8505 | Cell Signaling Technology, Danvers, MA, USA | 18 | 1:1000 |
| Rabbit anti-human claudin-1 monoclonal antibody [D5H1D] | 13255 | Cell Signaling Technology, Danvers, MA, USA | 20 | 1:1000 |
| Rabbit anti-human zonula occludens-2 polyclonal antibody (ZO-2) | 2847 | Cell Signaling Technology, Danvers, MA, USA | 150 | 1:1000 |
| Rabbit anti-human β-actin monoclonal antibody | 4970 | Cell Signaling Technology, Danvers, MA, USA | 45 | 1:1000 |
| Mouse anti-human E-cadherin monoclonal antibody [NCH-38] | M3612 | Dako, Carpinteria, CA, USA | 120 | 1:1000 |
| HRP-conjugated goat anti-rabbit IgG antibody | 7074 | Cell Signaling Technology, Danvers, MA, USA | - | 1:10000 |
| HRP-conjugated goat anti-mouse IgG antibody | 7076 | Cell Signaling Technology, Danvers, MA, USA | - | 1:10000 |

**Movie 1.** Video image showing time-lapse differential interference contrast microscopy of the leading-edge cells of the explant culture in normal KCM. Partial backward movements were observed in the lower-left region of the migrating edge from 2 h to 4 h.

**Movie 2.** Video image showing time-lapse differential interference contrast microscopy of the leading-edge cells of the explant culture in KCM-Ri.
